# Identification and validation of m^6^A RNA methylation and ferroptosis-related biomarkers in sepsis: transcriptome combined with single-cell RNA sequencing

**DOI:** 10.3389/fimmu.2025.1543517

**Published:** 2025-03-07

**Authors:** Jinshuai Lu, Jianhao Wang, Kun Han, Yuxia Tao, Jiyi Dong, Xiaoyu Pan, Xiaolan Wen

**Affiliations:** Department of Emergency, People’s Hospital of Xinjiang Uygur Autonomous Region, Urumqi, China

**Keywords:** sepsis, *N* ^6^-methyladenosine, ferroptosis, biomarkers, single-cell RNA sequencing

## Abstract

**Background:**

Sepsis, a systemic inflammatory response syndrome triggered by infection, is associated with high mortality rates and an increasing global incidence. While *N* ^6^-methyladenosine (m^6^A) RNA methylation and ferroptosis are implicated in inflammatory diseases, their specific genes and mechanisms in sepsis remain unclear.

**Methods:**

Transcriptomic datasets of sepsis, along with m^6^A-related genes (m^6^A-RGs) and ferroptosis-related genes (FRGs), were sourced from public databases. Differentially expressed genes (DEGs) were identified between the sepsis and control groups, and m^6^A-RGs were analyzed through weighted gene co-expression network analysis (WGCNA) to uncover m^6^A module genes. These were then intersected with DEGs and FRGs to identify candidate genes. Biomarkers were identified using two machine learning methods, receiver operating characteristic (ROC) curves, and expression validation, followed by the development of a nomogram. Further in-depth analyses of the biomarkers were performed, including functional enrichment, immune infiltration, drug prediction, and molecular docking. Single-cell analysis was conducted to identify distinct cell clusters and evaluate biomarker expression at the single-cell level. Finally, reverse transcription–quantitative PCR (RT-qPCR) was employed to validate biomarker expression in clinical samples.

**Results:**

DPP4 and TXN were identified as key biomarkers, showing higher expression in control and sepsis samples, respectively. The nomogram incorporating these biomarkers demonstrated strong diagnostic potential. Enrichment analysis highlighted their involvement in spliceosome function and antigen processing and presentation. Differential analysis of immune cell types revealed significant correlations between biomarkers and immune cells, such as macrophages and activated dendritic cells. Drug predictions identified gambogenic acid and valacyclovir as potential treatments, which were successfully docked with the biomarkers. Single-cell analysis revealed that the biomarkers were predominantly expressed in CD4^+^ memory cells, and CD16^+^ and CD14^+^ monocytes. The expression of DPP4 was further validated in clinical samples.

**Conclusions:**

DPP4 and TXN were validated as biomarkers for sepsis, with insights into immune infiltration and therapeutic potential at the single-cell level, offering novel perspectives for sepsis treatment.

## 1 Background

According to the 2016 *Third International Consensus Definitions for Sepsis and Septic Shock*, sepsis is described as “a syndrome of life-threatening organ dysfunction resulting from an abnormal host response to infection” (1). Characterized by high morbidity and mortality, sepsis remains a leading cause of death in modern intensive care units, contributing significantly to rising healthcare costs (2). The 2020 Global Burden of Disease report indicates that approximately 49 million individuals experience sepsis annually, with 11 million fatalities, accounting for approximately 20% of global mortality (3, 4). Notably, 90% of sepsis-related deaths occur in Asia and Africa (5). In 2017, the World Health Organization (WHO) recognized sepsis as a top-priority public health issue (6). Sepsis can result from trauma, severe burns, infections, major surgery, and other causes. Its pathogenesis is multifaceted, involving imbalances in inflammatory responses, immune dysfunction, mitochondrialdamage, coagulation abnormalities, neuroendocrine-immune network disruptions, endoplasmic reticulum stress, autophagy,and other pathophysiological mechanisms (7). Immunosuppression has emerged as a key factor contributing to sepsis mortality (8). Disruption of immune homeostasis triggers sepsis-induced immunosuppression, characterized by the release of anti-inflammatory cytokines, aberrant death of immune effector cells, unchecked proliferation of immunosuppressive cells, and the upregulation of immune checkpoints (9). Preclinical studies have shown that reversing immune dysfunction and enhancing host resistance can be achieved by targeting immunosuppression, particularly through immune checkpoint inhibitors. While antibiotics, fluid resuscitation, and organ support therapies are commonly used, they have limited impact on patient prognosis. Therefore, understanding the pathological role of sepsis-induced immunosuppression and identifying novel biomarkers is crucial for improving prevention and treatment strategies.

*N* ^6^-Methyladenosine (m^6^A) methylation is an epigenetic modification that primarily affects RNA molecules, including mRNA, lncRNA, and circRNA (10). This modification regulates gene expression by influencing RNA stability and fate. m^6^A has been shown to affect the half-life of mRNA in the cytoplasm, with clustered m^6^A sites promoting mRNA degradation (11). Recent studies suggest that m^6^A modification plays a role in various biological processes, including tumorigenesis, immune responses to viral infections, and several inflammatory diseases (12). Specifically, the heterogeneity of sepsis may be linked to m^6^A regulation (13), with analysis of m^6^A regulatory factors in sepsis revealing their involvement in sepsis development, immune cell infiltration, and inflammation (14). Ferroptosis, a recently recognized form of regulated cell death, is iron-dependent and results from an imbalance between reactive oxygen species (ROS) production and degradation (15). Ferroptosis is implicated in the pathogenesis and progression of numerous diseases, and its signaling pathways offer promising druggable targets (16). The potential of ferroptosis inhibitors in sepsis treatment has been increasingly demonstrated (17). Understanding sepsis pathogenesis and developing drugs that target these underlying mechanisms are essential for advancing treatment strategies in this field.

Single-cell RNA sequencing (scRNA-seq) is a high-throughput technique that provides detailed insights into the transcriptomes of individual cells (18). By examining cells at the single-cell level, scRNA-seq enhances data resolution and precision, revealing the distribution and functional status of diverse cell types within tissues. Advances in scRNA-seq technology and data analysis methods have facilitated the identification of molecular characteristics in immune cell populations within sepsis, offering a novel approach to discovering functional biomarkers (19).

This study leveraged bioinformatics tools to identify m^6^A- and ferroptosis-related biomarkers in sepsis using publicly available transcriptome data. Following this, various analyses were performed on these biomarkers, and their expression at the single-cell level was explored, providing a theoretical foundation for understanding the mechanisms and improving the diagnosis of sepsis.

## 2 Methods

### 2.1 Source of data

Three sepsis transcriptomic datasets were retrieved from the Gene Expression Omnibus (GEO) database (https://www.ncbi.nlm.nih.gov/geo/). The GSE65682 dataset (GPL13667) contained human blood samples, with a sepsis:control ratio of 760:42, serving as the training set. GSE13904 (GPL570), comprising human blood samples with a sepsis:control ratio of 52:18, was used as the validation set. The training dataset used in this study primarily originated from an adult population, whereas the validation dataset was sourced from a pediatric population. Due to time and computational resource constraints, it was not possible to mix the two datasets and reanalyze them at this stage. Additionally, the GSE167363 (GPL24676) dataset, consisting of peripheral blood mononuclear cells (PBMCs) with a sepsis:control ratio of 2:10, was employed for single-cell analysis (20). Furthermore, 834 ferroptosis-related genes (FRGs) were extracted from the FerrDb database (http://www.zhounan.org/ferrdb/current/) (**Supplementary Table S1**), and 17 m^6^A-related genes (m^6^A-RGs) were obtained from a published study (14) (**Supplementary Table S2**).

### 2.2 Selection of candidate genes

First, differential expression analysis was performed on the training set to identify differentially expressed genes (DEGs) between sepsis and control samples. DEGs were selected with |log2Fold Change (FC)| > 0.5 and p < 0.05 using the “limma” package (v 3.58.1) (21). Volcano map and heat map were generated using the “ggplot2” (v 3.3.6) (22) and “ComplexHeatmap” (v 2.14.0) (23) packages for DEG visualization. The volcano plot displayed the number of DEGs and the top 10 up- and downregulated DEGs, ranked by |log2FC| in descending order, while the heatmap showed the distribution and expression of the top 10 up- and downregulated DEGs. Next, to assess the variation of m^6^A-RGs between sepsis and control samples in the training set, single-sample gene set enrichment analysis (ssGSEA) was conducted using the “GSVA” package (v 1.50.0) (24). Each m^6^A-RG was scored, and the Wilcoxon test was used to compare the ssGSEA scores of m^6^A-RGs between sepsis and control samples (p < 0.05). Subsequently, to identify module genes highly correlated with ssGSEA scores, weighted gene co-expression network analysis (WGCNA) was performed. Prior to WGCNA, the presence of outlier samples and sample clustering were checked using the goodSamplesGenes and hclust functions from the “WGCNA” package (v 1.72-5) (25). At the same time, the median absolute deviation (MAD) of each gene was calculated, and the genes with MAD values in the bottom 25% were removed to screen out genes with larger expression changes. The optimal soft threshold (power) was determined based on a scale-free fit index (R^2^ = 0.85) and the mean connectivity approach (power = 0). Genes were grouped into different modules based on this power, with a minimum of 200 genes per module to ensure the modules had clear biological meaning and statistical reliability. The module fusion threshold of 0.35 was set to merge highly similar modules. Pearson’s correlation was used to examine the relationships between module eigengene (ME) scores and ssGSEA scores of m^6^A-RGs. Gene modules with the highest positive and negative correlations (|correlation (R)| > 0.3, p < 0.05) with ssGSEA scores were selected as key modules, with R > 0.3 being a commonly used threshold in WGCNA (26), and the genes within these key modules were regarded as m^6^A module genes. Finally, the DEGs, m^6^A module genes, and FRGs were intersected to identify candidate genes using the “ggvenn” package (v 0.1.10) (DOI: 10.32614/CRAN.package.ggvenn).

### 2.3 Enrichment analyses and protein–protein interaction of candidate genes

Potential biological functions and pathways of candidate genes were explored using the “clusterProfiler” package (v 4.7.1.3) (27) through Gene Ontology (GO) and Kyoto Encyclopedia of Genes and Genomes (KEGG) analyses (p < 0.05). The top 5 significantly enriched terms in each GO category [biological process (BP), molecular function (MF), and cellular component (CC)] and the top 5 KEGG pathways, ranked by ascending p-values, were presented. To investigate protein-level interactions of the candidate genes, they were uploaded to the STRING database (http://string-db.org/) with parameters (species = human, confidence > 0.9). The protein–protein interaction (PPI) network was constructed using “Cytoscape” (v 3.10.2) (28), and interacting candidate genes were extracted for further analysis.

### 2.4 Identification and validation of biomarkers

Two machine learning algorithms, least absolute shrinkage and selection operator (LASSO) and support vector machine recursive feature elimination (SVM-RFE), were used to identify candidate biomarkers. The “glmnet” package (v 4.1-8) (29) was applied to create the LASSO regression model, and model optimization was guided by 10-fold cross-validation, where lambda was the regularization parameter that determined the strength of L1 regularization. In particular, lambda.min corresponds to the value that produces the minimum cross-validation error, and lambda.1se provides the most concise model within a standard error range of the minimum error. In this discovery phase, we prioritize the retention of biologically reasonable candidate genes, and therefore, the genes were selected according to lambda.min. For SVM-RFE, the “caret” package (v 6.0-94) (30) was utilized to evaluate the importance of genes. The accuracy rate of each iteration was computed, and genes were selected when accuracy reached its highest value. The overlap of genes selected by both algorithms was considered as candidate biomarkers. To assess the diagnostic performance of the candidate biomarkers, receiver operating characteristic (ROC) curves were generated for the biomarkers in the GSE65682 and GSE13904 datasets using the “pROC” package (v 1.18.5) (31). A candidate biomarker with an area under the curve (AUC) > 0.7 was deemed to have high diagnostic accuracy and was forwarded for further analysis. Lastly, the expression levels of the candidate biomarkers were compared between the sepsis and control groups in the GSE65682 and GSE13904 datasets using the Wilcoxon test (p < 0.05). Biomarkers exhibiting differential expression between the two groups and consistent expression trends across both datasets were identified as potential diagnostic biomarkers.

### 2.5 Nomogram establishment

After identifying biomarkers, the role of these biomarkers in diagnosing sepsis was quantified by creating a diagnostic nomogram using the “rms” package (v 6.5-0) (32), based on the expression levels of the biomarkers. The nomogram included both individual and total points, where the total points indicated the patient’s morbidity risk for sepsis. Higher total points corresponded to a higher likelihood of sepsis. To evaluate the nomogram’s effectiveness, a calibration curve, generated with the “rms” package, the Hosmer–Lemeshow (HL) test, and the ROC curve plotted by the “pROC” package (v 1.18.5) were employed. A calibration curve that closely matched the ideal curve, a p-value from the HL test greater than 0.05, and an AUC of the ROC curve greater than 0.7 indicated that the nomogram had strong predictive capability.

### 2.6 Correlation and function analyses of biomarkers

To elucidate the correlation and function of the biomarkers, Spearman’s correlation coefficients were calculated using the “cor” function in R (v 4.2.2) for biomarkers in the training set, where |R| > 0.3 and p < 0.05 were considered significant correlations. Additionally, Gene Set Enrichment Analysis (GSEA) was conducted on the biomarkers to identify the biological pathways they were involved in. Spearman’s correlation coefficients between each biomarker and all genes in the sepsis samples from the training set were calculated, and the coefficients were ranked in descending order to create gene lists for each biomarker. GSEA was performed using the “GSVA” (v 1.50.0) package with thresholds of p < 0.05, |normalized enrichment score (NES)| > 1, and false discovery rate (FDR) < 0.25. The reference gene set “c2.cp.kegg.v7.4.symbols.gmt” was imported from the Molecular Signatures Database (MSigDB, https://www.gsea-msigdb.org/gsea/msigdb), and the top 5 enriched results, ranked by FDR in ascending order, were visualized using the “enrichplot” package (v 1.22.0) (33).

### 2.7 Analysis of immune infiltration

Since the immune system plays a key role in the development of sepsis, the ssGSEA scores of 28 types of immune cells (34) were calculated using the “GSVA” package (v 1.50.0) to assess immune cell infiltration levels in the training set. Differential immune cell infiltration between the sepsis and control groups was identified using the Wilcoxon test (p < 0.05). The “psych” package (v 2.2.5) (35) was used to examine correlations between differential immune cells and biomarkers (|R| > 0.3, p < 0.05).

### 2.8 Drug prediction and molecular docking

To predict drugs targeting the biomarkers, the Drug–Gene Interaction Database (DGIdb; www.dgidb.org) was used to identify potential drugs for sepsis based on the biomarkers. A drug prediction network was constructed using “Cytoscape” (v 3.10.2). Furthermore, to explore biomarker–drug interactions in more detail, the top 3 drugs with the highest interaction scores for each biomarker were selected for molecular docking. The three-dimensional structures of the drugs and biomarkers were retrieved from the PubChem database (https://pubchem.ncbi.nlm.nih.gov/) and the Protein Data Bank (PDB; https://rcsb.org/), respectively. Molecular docking was performed using the CB-Dock2 website (https://cadd.labshare.cn/), and the Vina scores were calculated using AutoDock Vina (v 1.2.0) (36) to assess the binding energy between the drugs and biomarkers. Lower Vina scores indicated stronger binding between the drugs and biomarkers.

### 2.9 Single-cell data processing

For single-cell analysis, the “Seurat” package (v 5.0.1) (37) and the GSE167363 dataset were used. First, during single-cell quality control (QC), the number of detected genes per cell (nFeature_RNA), total RNA counts per cell (nCount_RNA), and the percentage of mitochondrial gene expression (percent.mt) were assessed. Cells were excluded if they contained fewer than 200 genes or if they were represented by fewer than three genes. Cells with nFeature_RNA >200 and <6,000, nCount_RNA >500 and <10,000, and percent.mt < 10% were selected for subsequent analysis. Next, the NormalizeData function was used to normalize the data, and the top 2,000 highly variable genes were identified using the FindVariableFeatures function. These genes were exported for further analysis. To reduce the dimensionality of the data, principal component analysis (PCA) was performed using the RunPCA function, and the results were visualized and filtered by the JackStrawPlot and Elbowplot functions (p < 0.05). The principal components (PCs) from PCA were then passed to the FindNeighbors and FindClusters functions to conduct unsupervised clustering. t-Distributed stochastic neighbor embedding (t-SNE) clustering was performed using the RunTSNE function (resolution = 0.6) to group the data, and cell clusters were identified.

### 2.10 Cell annotation and expression verification in cell clusters

To annotate the cell clusters obtained from t-SNE, marker genes extracted from a published paper (38) (**Table 1**) were used to classify the cell clusters into different cell types. The Dotplot function in the “Seurat” package (v 5.0.1) was used to display the expression of marker genes for each cell cluster, with highly expressed markers highlighted. After determining the cell types, the distribution of cell types between the sepsis and control groups was examined. Finally, the expression and distribution of biomarkers in each cell type were explored and visualized using the FeaturePlot and VlnPlot functions.

**Table 1:** Cell makers.

Cell type	Marker genes
B cells	"MS4A1", "CD79A","CD37"
CD16+ and CD14+ monocytes	"CD68", "CD14","S100A12"
CD4+ memory cells	"IL7R", "CD27","CCR7"
CD8+ T cells	"CD8A", "CD8B"
Megakaryocyte progenitors	"PF4", "PPBP", "PLA2G12A"
Natural killer (NK) cells	"CD160","NKG7", "GNLY"

### 2.11 Experimental validation

RT-qPCR was performed to assess the expression of biomarkers in clinical samples from patients with sepsis and healthy controls. A total of 10 whole human blood samples were collected from the People’s Hospital of Xinjiang Uygur Autonomous Region (sepsis:control = 5:5). Informed consent was obtained from all participants, and the study was approved by the Ethics Committee of People’s Hospital of Xinjiang Uygur Autonomous Region. A portion of the collected samples was used for transcriptome sequencing, while the remaining samples were stored at −80°C for subsequent RT-qPCR experiments. Total RNA was extracted using TRIzol reagent, and reverse transcription was performed to synthesize complementary DNA (cDNA). Quantitative PCR was conducted using cDNA as the template with primers listed in **Supplementary Table S7**, and gene expression levels were quantified using the 2^−ΔΔCt^ method. The GraphPad Prism (v 5.0) (39) software was used for result visualization.

### 2.12 Statistical analysis

All bioinformatics analyses were performed using the R programming software (v 4.2.2). The Wilcoxon test was applied to compare differences between the two groups, and the t-test was used to analyze differences in RT-qPCR data. A p-value of <0.05 was considered statistically significant.

## 3 Results

### 3.1 A total of 85 candidate genes were identified

**Figure 1:**
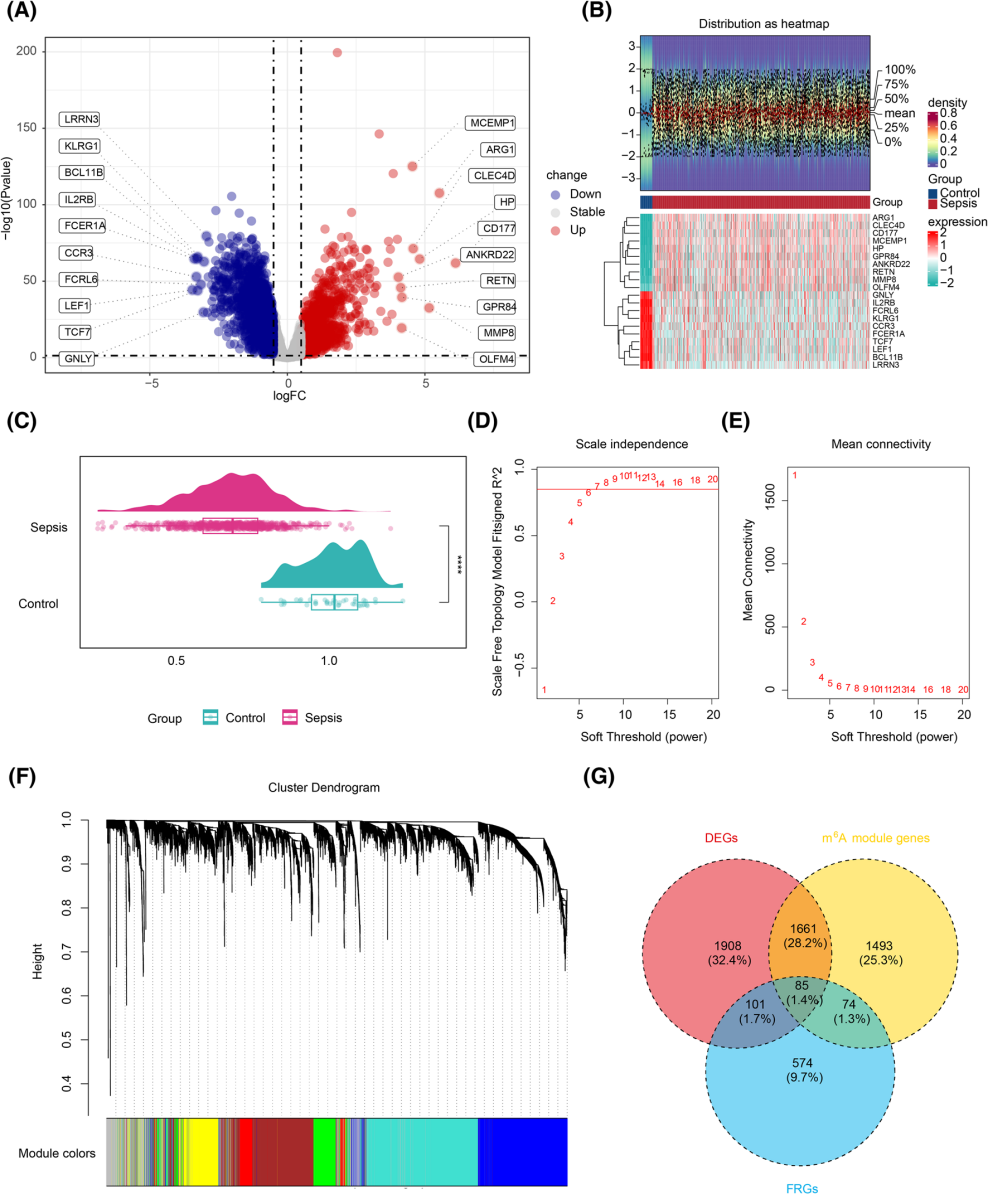
Screening for biomarkers related to m^6^A RNA methylation and ferroptosis. **(A)** Volcano plot of the differential analysis of the GSE65682 gene set, highlighting significant variations in gene expression. **(B)** Expression density heatmap and expression heatmap. The expression density heatmap at the upper part of the figure shows kernel density estimation of expression distribution for each gene, with red colors indicating higher density. At the bottom of the figure is the expression heatmap. **(C)** ssGSEA score raincloud plot, with the ssGSEA score as the abscissa. ****, p < 0.0001. **(D)** Scale-free fitting exponent analysis with multiple soft threshold powers. **(E)** The average connectivity analysis with multiple soft threshold powers. **(F)** Dendrogram of genes based on clustering using the topological overlap matrix measure, with the color band displaying results obtained from automatic single-block analysis. **(G)** Venn diagram of the intersection of differentially expressed genes, m^6^A-related genes, and iron-related death genes. m^6^A, *N* ^6^-methyladenosine; ssGSEA, single-sample gene set enrichment analysis.

Differential expression analysis identified 3,755 DEGs in the training set, including 1,524 upregulated and 2,231 downregulated genes (**Figure 1A**). The top 10 up- and downregulated DEGs were labeled, and their expression and distribution are presented in **Figure 1B**. Prior to performing WGCNA, the ssGSEA scores for m^6^A-RGs in the training set were significantly elevated in the control group (p < 0.05) (**Figure 1C**), confirming the suitability of proceeding with WGCNA. After excluding one outlier sample, the remaining training set samples were clustered (**Supplementary Figure S1A**). The optimal “power” threshold was determined to be seven based on the scale-free fit index (R^2^ = 0.85) and the mean connectivity approach (**Figures 1D, E**), resulting in the construction of a gene co-expression network. This network revealed that genes from six modules were successfully clustered, excluding the gray module (**Figure 1F**). Correlation analysis identified MEbrown as the most positively correlated module (R = 0.61, p < 0.05) and MEblue as the most negatively correlated (R = −0.73, p < 0.05) (**Supplementary Figure S1B**), leading to the identification of 3,313 genes in these modules as m^6^A-related genes. A subsequent intersection of these 3,313 m^6^A genes, 3,755 DEGs, and 834 FRGs yielded 85 candidate genes (**Figure 1G**).

### 3.2 Enrichment results and PPI network of candidate genes

**Figure 2:**
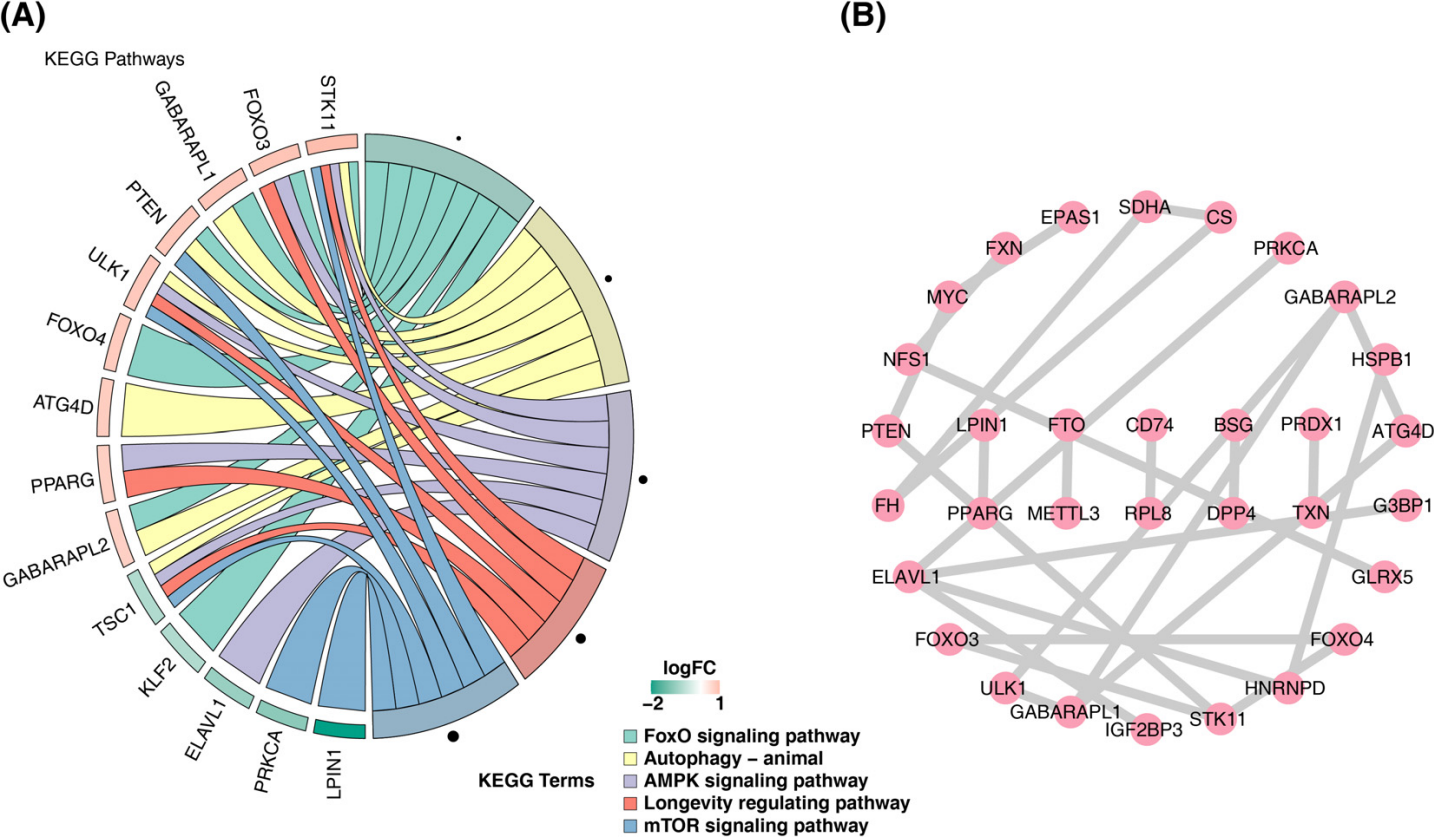
Enrichment results and PPI network of candidate genes. **(A)** Diagram of the KEGG analysis. The left half shows the enriched gene names, and the shade represents the logFC magnitude. The right half shows the enriched functional pathways. **(B)** Protein interaction network diagram. PPI, protein–protein interaction; KEGG, Kyoto Encyclopedia of Genes and Genomes.

GO enrichment analysis of the candidate genes identified 754 entries, comprising 643 BPs, 36 CCs, and 75 MFs (**Supplementary Table S3**). The most significantly enriched BPs included myeloid cell differentiation (GO:0030099) and mononuclear cell differentiation (GO:1903131), key CCs included mitochondrial matrix (GO:0005759) and iron–sulfur cluster assembly complex (GO:1990229), and prominent MFs included DNA-binding transcription factor binding (GO:0140297) and RNA polymerase II-specific DNA-binding transcription factor binding (GO:0061629) (**Supplementary Figure S2**), indicating that the candidate genes were largely involved in differentiation and gene expression regulation. Additionally, 22 KEGG pathways were enriched (**Supplementary Table S4**), including the FoxO signaling and mTOR signaling pathways (**Figure 2A**). The PPI network revealed protein interactions among 32 candidate genes, including GABARAPL1, ULK1, and FOXO3 (**Figure 2B**), which were thus selected as potential biomarkers.

### 3.3 TXN and DPP4 were identified as biomarkers

**Figure 3:**
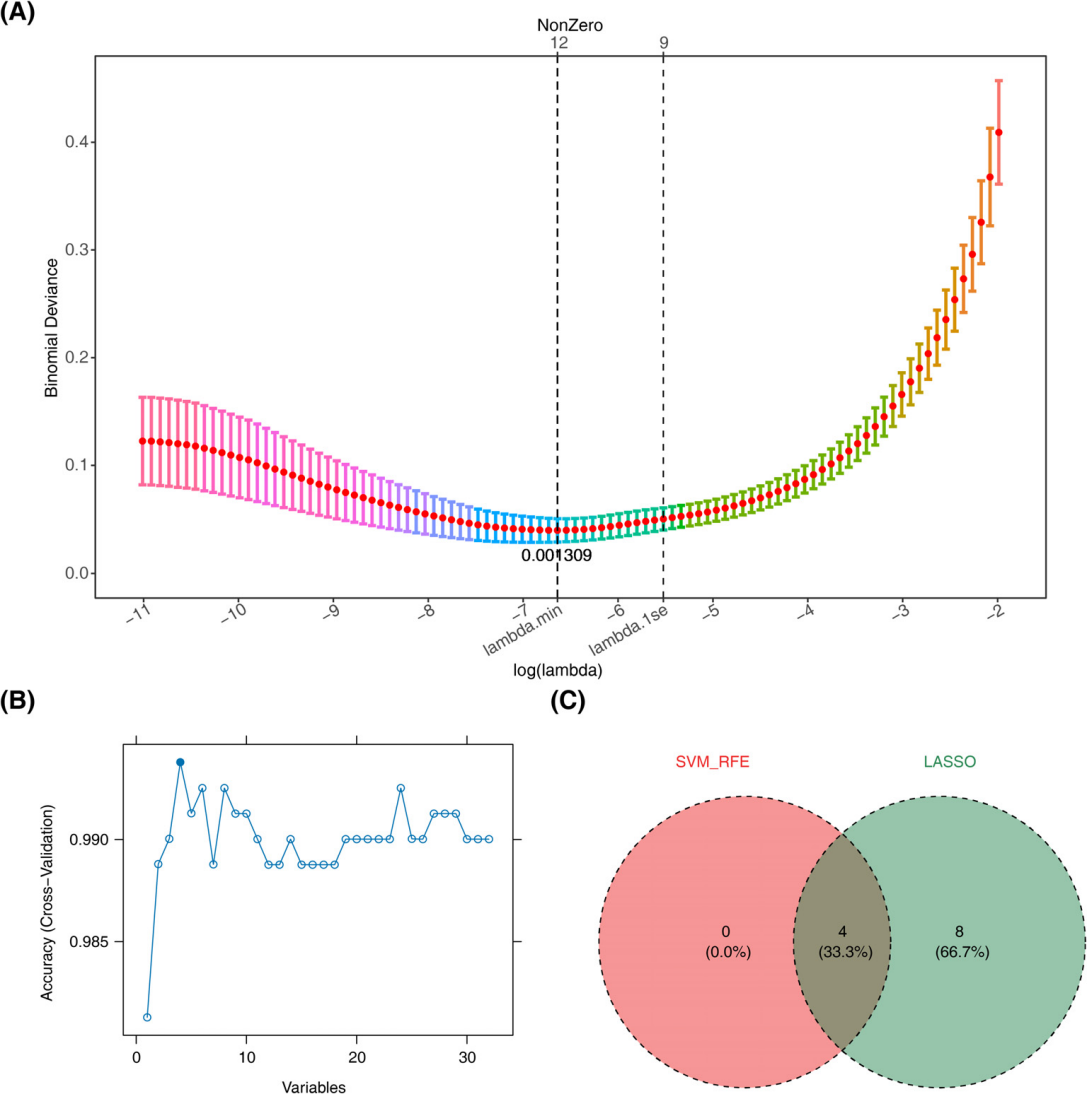
Machine learning screening. **(A)** The process of selecting the optimal parameter λ for the LASSO regression model using cross-validation. **(B)** Results of the SVM-RFE algorithm. **(C)** Venn diagram of genes investigated by SVM and LASSO. LASSO, least absolute shrinkage and selection operator; SVM-RFE, support vector machine recursive feature elimination.

**Figure 4:**
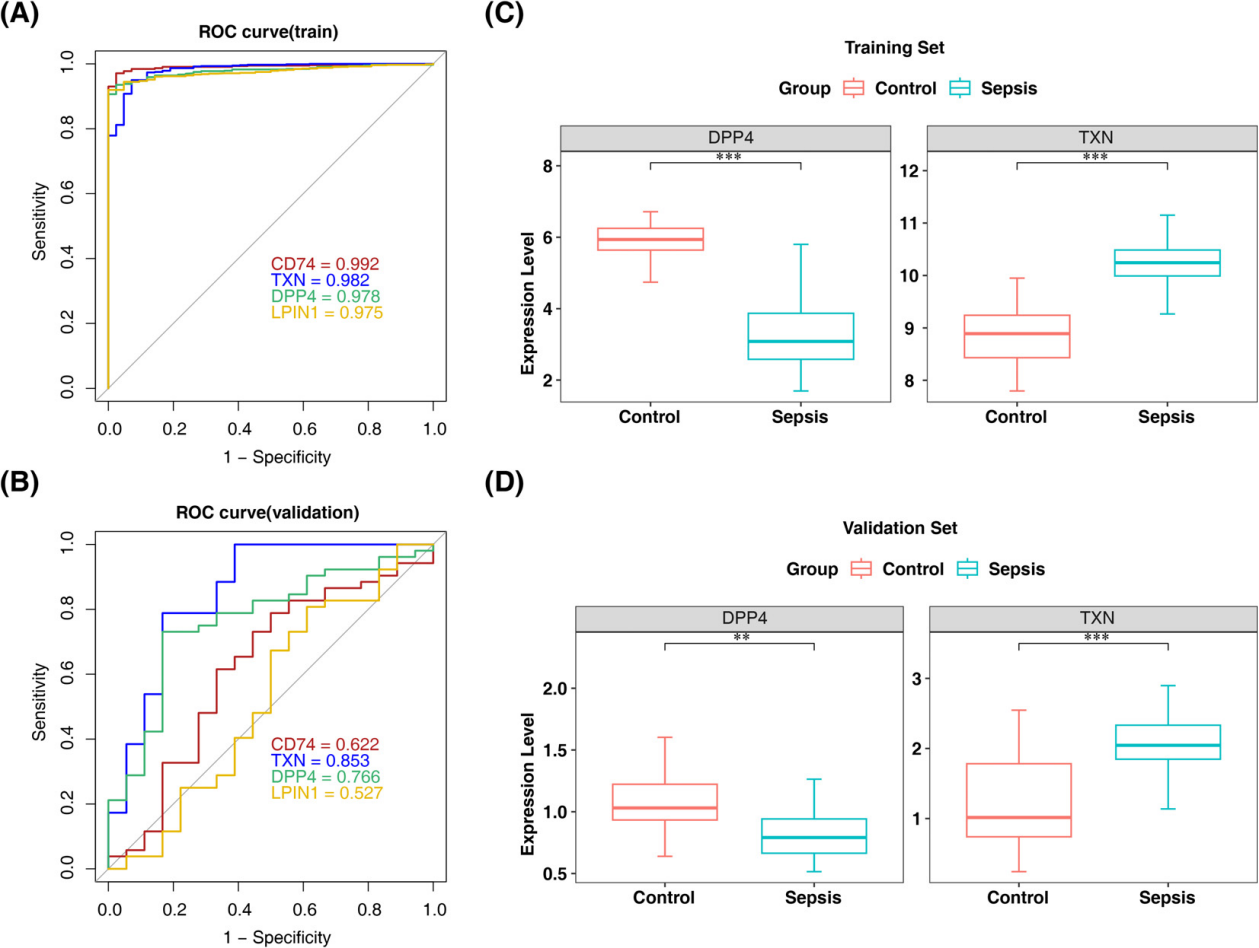
ROC curve and expression level validation for screening biomarkers. **(A, B)** ROC curves of the training and validation sets of candidate biomarkers. **(C, D)** Analysis of expression levels in the training and validation sets of sepsis biomarkers. **, p < 0.01; ***, p < 0.001. ROC, receiver operating characteristic.

Subsequently, machine learning was applied to the 32 candidate genes. LASSO analysis identified 12 candidate biomarkers (TXN, PPARG, LPIN1, HSPB1, NFS1, FXN, IGF2BP3, SDHA, CS, CD74, DPP4, and ATG4D) at lambda.min = 0.001309 (**Figure 3A**, **Supplementary Figure S3**). Concurrently, SVM-RFE selected four candidate biomarkers (CD74, TXN, DPP4, and LPIN1) based on the highest model accuracy (**Figure 3B**, **Supplementary Table S5**), leading to the identification of four overlapping biomarkers (CD74, TXN, DPP4, and LPIN1) (**Figure 3C**). ROC curve analysis demonstrated AUC values >0.7 for TXN and DPP4 in both the training and validation sets, indicating their potential to effectively distinguish patients with sepsis (**Figures 4A, B**). Further expression analysis revealed that TXN and DPP4 were differentially expressed in both datasets (p < 0.05) and exhibited consistent trends (**Figures 4C, D**). Specifically, TXN was upregulated in the sepsis group, while DPP4 was elevated in the control group, positioning TXN and DPP4 as potential biomarkers.

### 3.4 A nomogram was developed and verified

**Figure 5:**
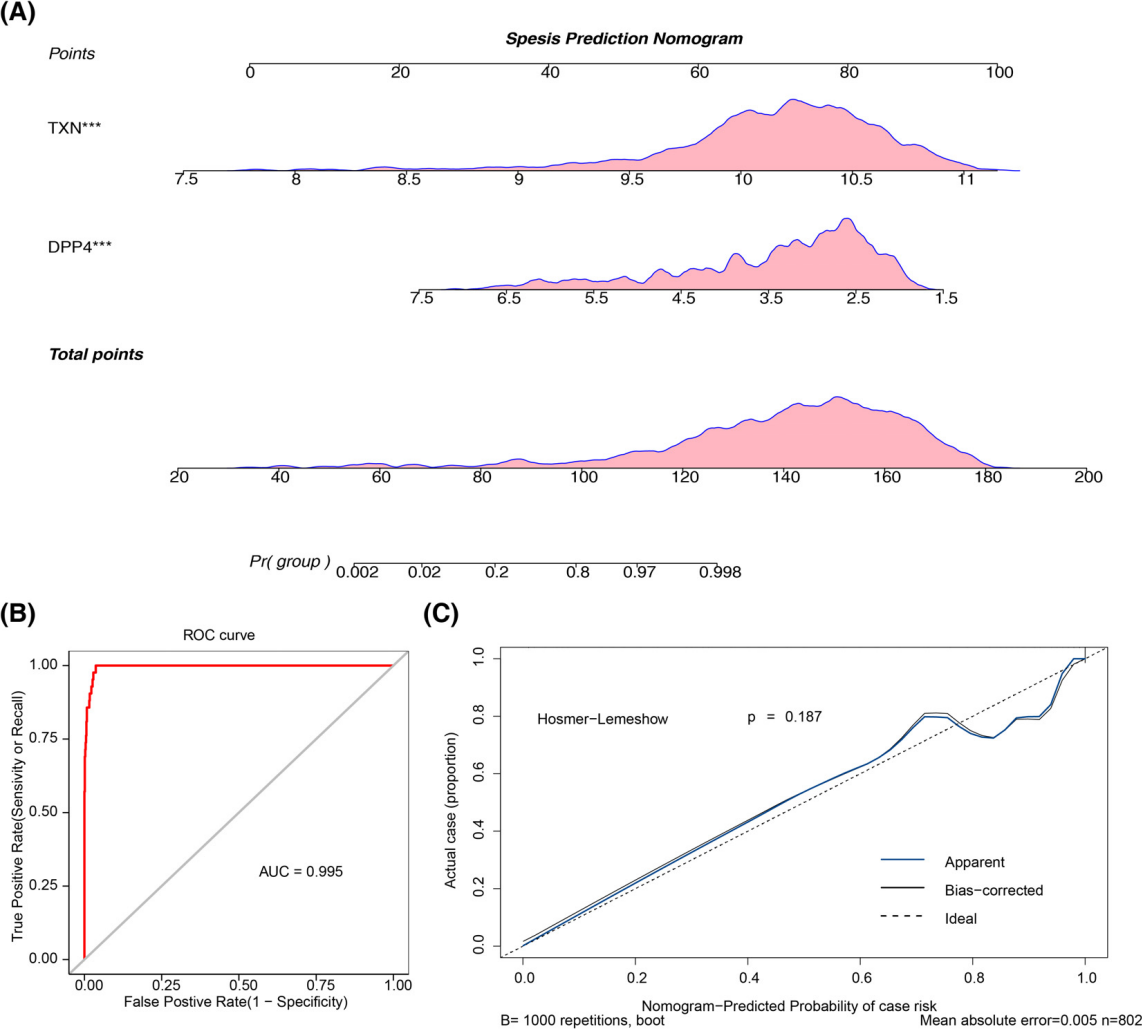
The construction and validation of the nomogram. **(A)** Sepsis prediction nomogram. The frequency distribution is shown above the axis. **(B)** ROC curve of the prediction model. **(C)** Calibration curve of the prediction model. ROC, receiver operating characteristic.

By utilizing the expression of two biomarkers, a nomogram was constructed to predict sepsis morbidity (**Figure 5A**), indicating that higher total points correlate with an increased sepsis risk. The nomogram’s predictive performance was subsequently assessed: the AUC of the ROC curve exceeded 0.9 (**Figure 5B**), and the apparent and ideal curves closely aligned in the calibration plot, with a p-value > 0.05 from the HL test (**Figure 5C**), collectively confirming the nomogram’s robust predictive ability.

### 3.5 Correlation and GSEA results of biomarkers

**Figure 6:**
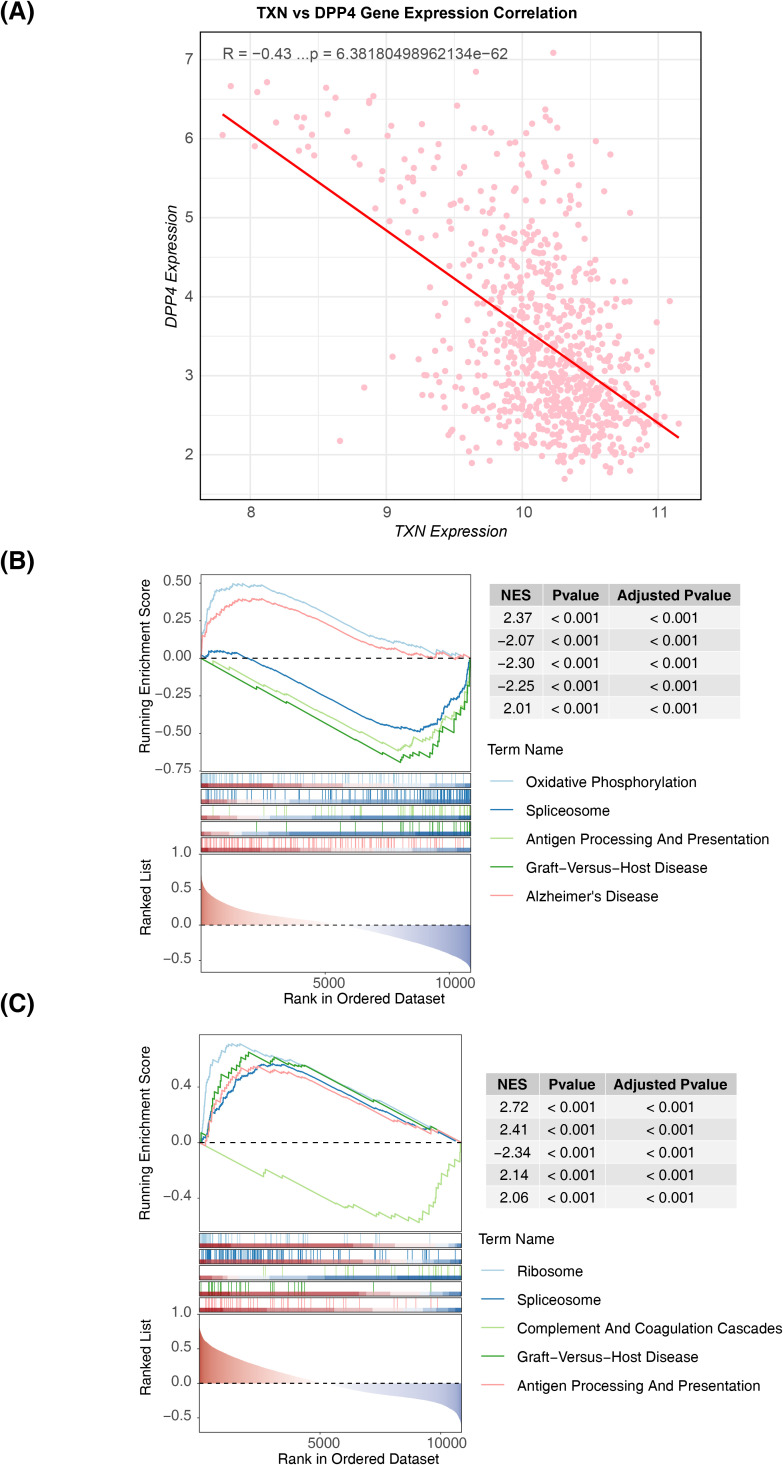
Correlation distribution map of biomarkers and GSEA functional enrichment map. **(A)** Scatter plot of the expression correlation between TXN and DPP4. **(B)** GSEA result map of TXN. **(C)** GSEA result map of DPP4. GSEA, Gene Set Enrichment Analysis.

Further correlation analysis and GSEA revealed the functions of these biomarkers. A significant negative correlation was observed between the two biomarkers (cor = −0.43, p < 0.05) (**Figure 6A**). GSEA identified that TXN was enriched in 39 pathways, including oxidative phosphorylation, spliceosome, and antigen processing and presentation (**Figure 6B**, **Supplementary Table S6**), while DPP4 was involved in 42 pathways such as ribosome biogenesis, spliceosome, and complement and coagulation cascades (**Figure 6C**, **Supplementary Table S6**). Notably, both biomarkers were co-enriched in pathways such as spliceosome and antigen processing and presentation, suggesting their close association with immune responses and gene expression regulation.

### 3.6 Results of immune infiltration

**Figure 7:**
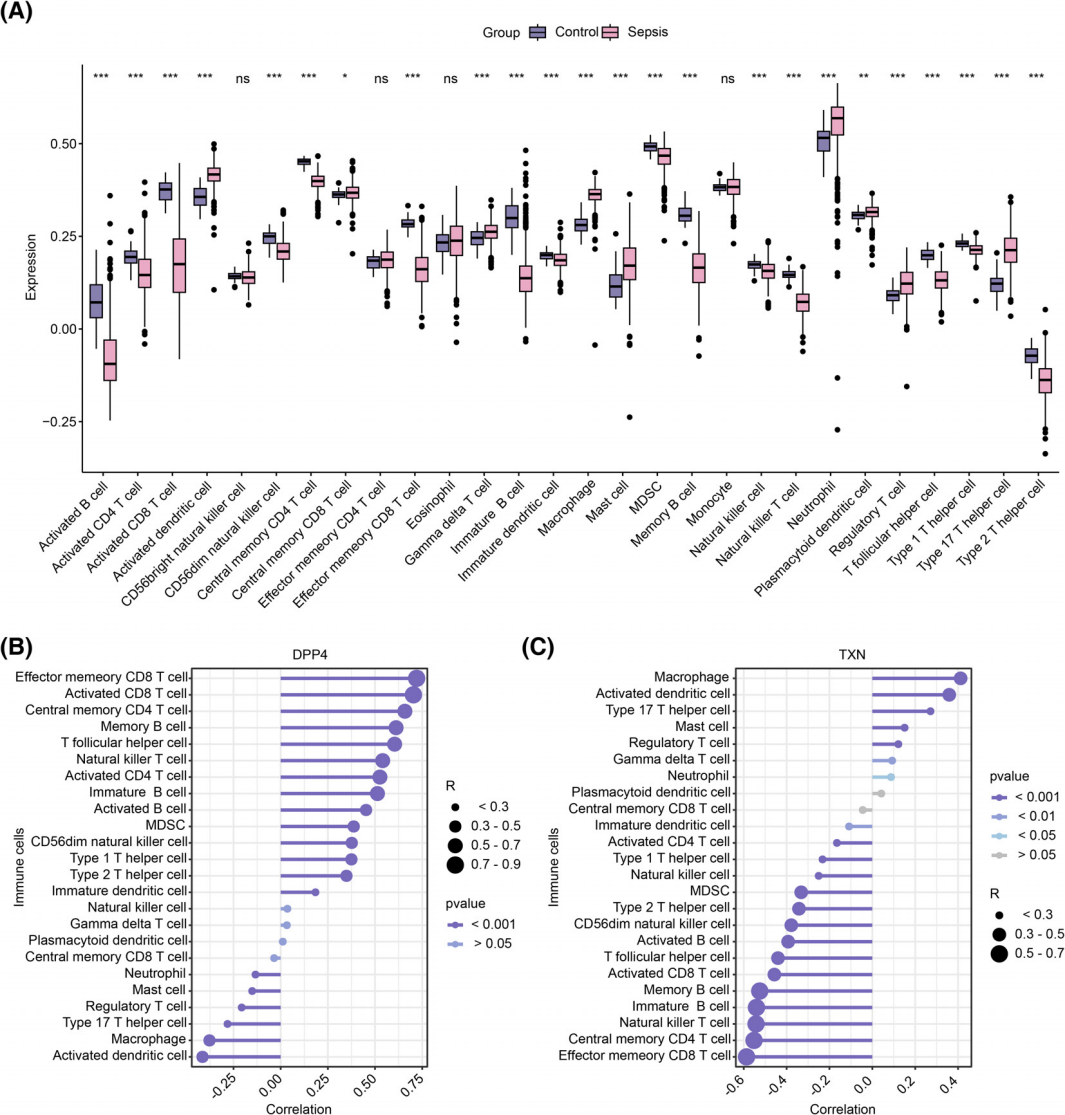
Immune infiltration analysis. **(A)** Box plot of differential immune cell infiltration. **(B)** Correlation between DPP4 and differential immune cells. ns represents no significance; *, p < 0.05; **, p < 0.01; ***, p < 0.001. **(C)** Correlation between TXN and differential immune cells. **(B, C)** The circle size represents the correlation size, and the line color shade represents the p-value size.

Immune cell infiltration levels across 28 immune cell types were compared between the sepsis and control groups in the training set (**Supplementary Figure S4**). Differential analysis revealed 24 immune cell types with significant differences in infiltration; activated dendritic cells, plasmacytoid dendritic cells, central memory CD8 T cells, gamma delta T cells, macrophages, mast cells, neutrophils, regulatory T cells, and type 17 T helper cells had higher infiltration levels in the sepsis group, whereas the remaining 15 cell types were more abundant in the control group (p < 0.05) (**Figure 7A**). Correlation analysis further showed that DPP4 exhibited strong positive correlations with effector memory CD8 T cells and activated CD8 T cells (R > 0.3, p < 0.05), while macrophages and activated dendritic cells were negatively correlated with DPP4 (R < −0.3, p < 0.05) and positively correlated with TXN (R > 0.3, p < 0.05). Conversely, effector memory CD8 T cells and central memory CD4 T cells showed a strong negative correlation with TXN (R < −0.3, p < 0.05) (**Figures 7B, C**).

### 3.7 The drug prediction network and molecular docking results

**Figure 8:**
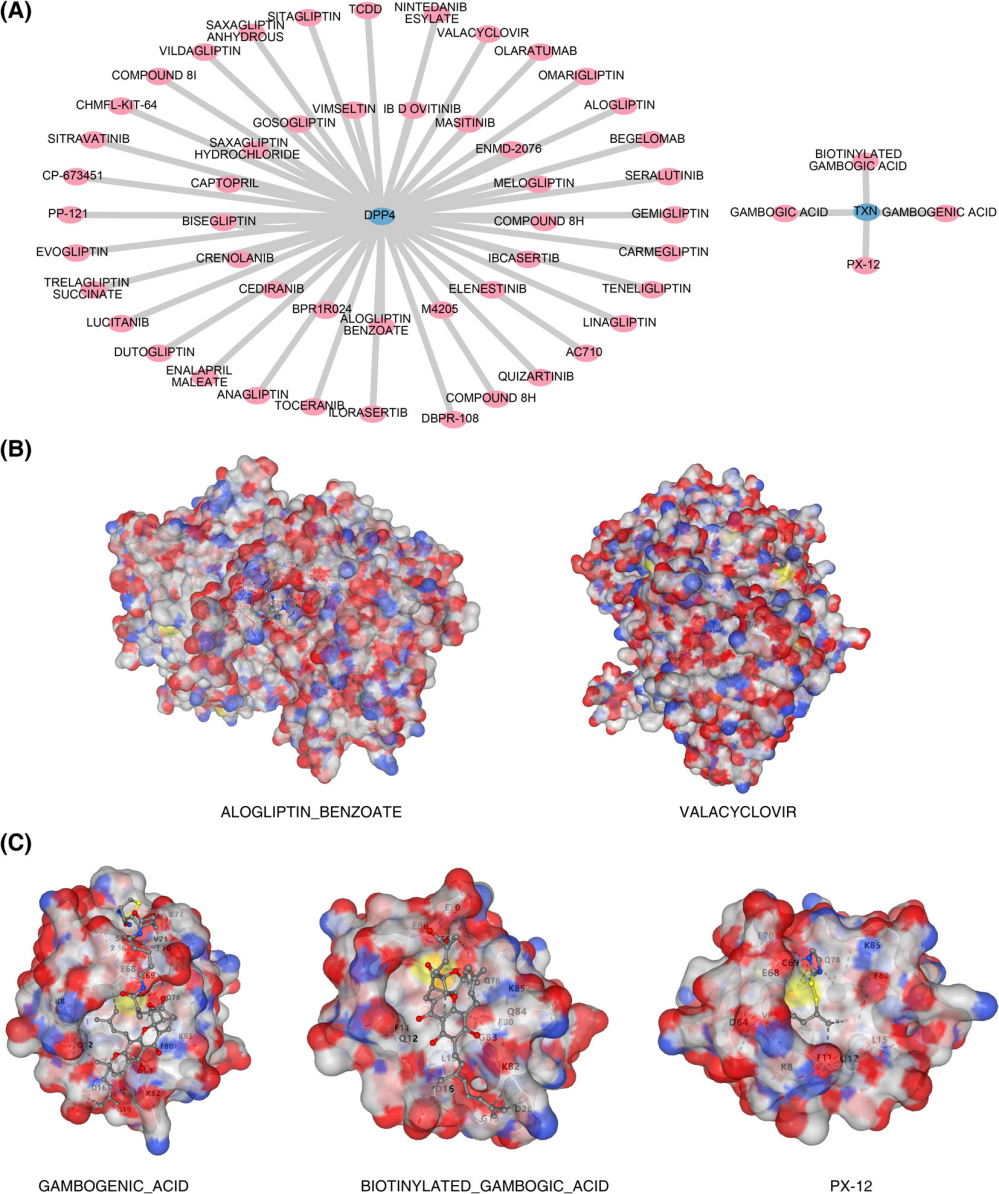
Biomarkers and drug prediction. **(A)** Drug–biomarker interaction network. **(B)** Results of docking between DPP4 and drug molecules. On the left is ALOGLIPTIN_BENZOATE, and on the right is VALACYCLOVIR. **(C)** Results of docking between TXN and drug molecules. On the left is BIOTINYLATED_GAMBOGIC_ACID, in the middle is GAMBOGENIC_ACID, and on the right is PX-12.

Based on drug prediction analysis, 50 drugs were identified to target DPP4, including alogliptin and begelomab, while four drugs were found to target TXN, such as biotinylated gambogic acid and gambogenic acid. No drug was predicted to target both biomarkers simultaneously (**Figure 8A**). Molecular docking of the top 3 drugs, selected based on their interaction scores with each biomarker, was then performed (**Figures 8B, C**). However, docking between dutogliptin and DPP4 yielded no successful binding. The highest Vina scores were observed between gambogenic acid and TXN (−6.7) and between valacyclovir and DPP4 (−6.7, **Table 2**), indicating a stronger binding affinity of these drugs to the respective biomarkers.

**Table 2:** Molecular docking scores.

Protein	Molecule	Vinascore
TXN	PX-12	-3.9
TXN	BIOTINYLATED_GAMBOGIC_ACID	-6.5
TXN	GAMBOGENIC_ACID	-6.7
DPP4	VALACYCLOVIR	-6.7
DPP4	DUTOGLIPTIN	NA
DPP4	ALOGLIPTIN_BENZOATE	-5.3

### 3.8 Identification of cell clusters and expression of biomarkers in single-cell data

**Figure 9:**
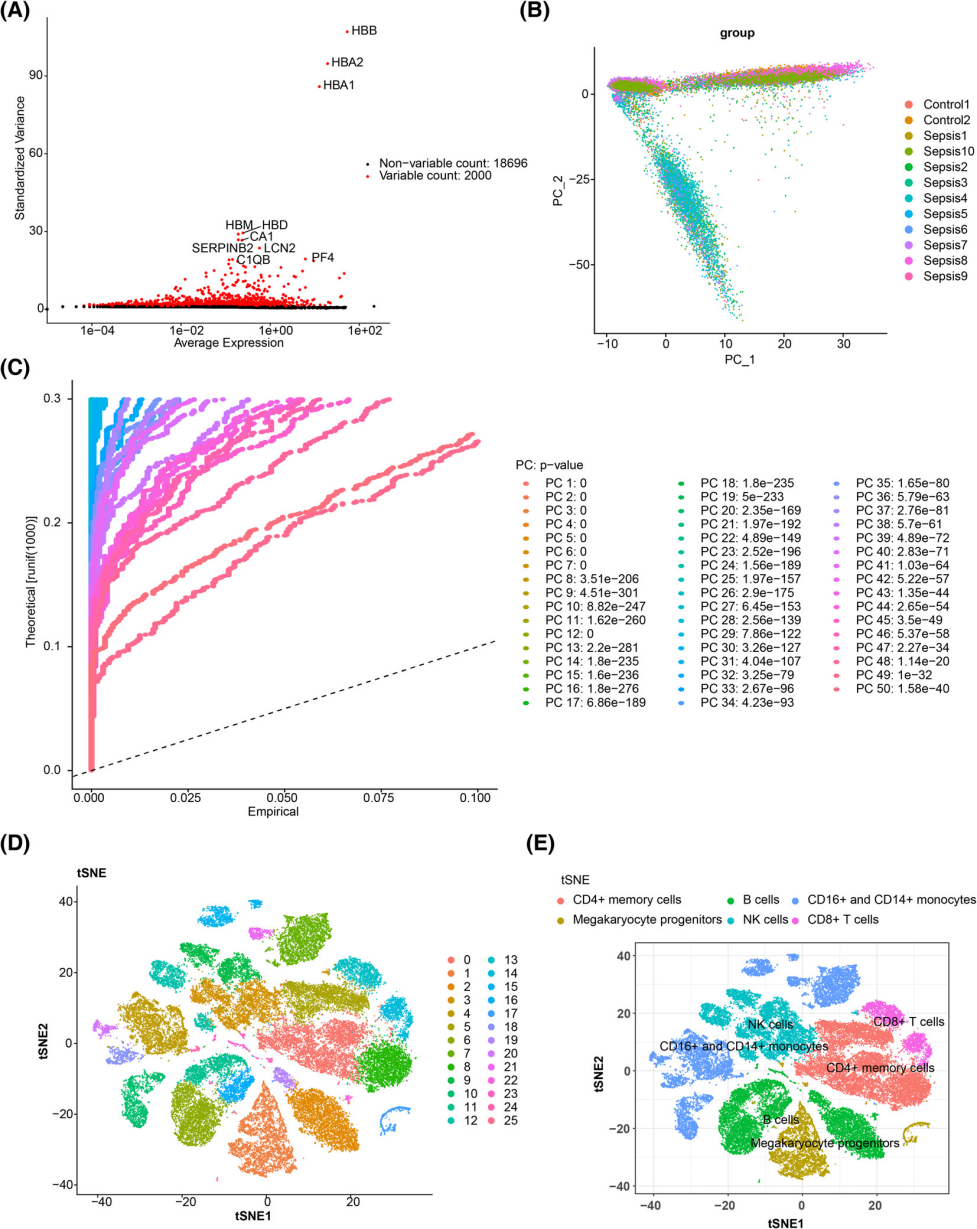
Single-cell sequencing analysis. **(A)** Screening of highly variable genes. The red color in the figure represents highly variable genes, and the top 10 most significantly variable genes are marked. **(B)** PCA sample cell distribution map. **(C)** JackStraw map. **(D)** Cell t-SNE clustering diagram. Different colors correspond to different clusters. **(E)** Cell annotation t-SNE diagram. PCA, principal component analysis; t-SNE, t-distributed stochastic neighbor embedding.

**Figure 10:**
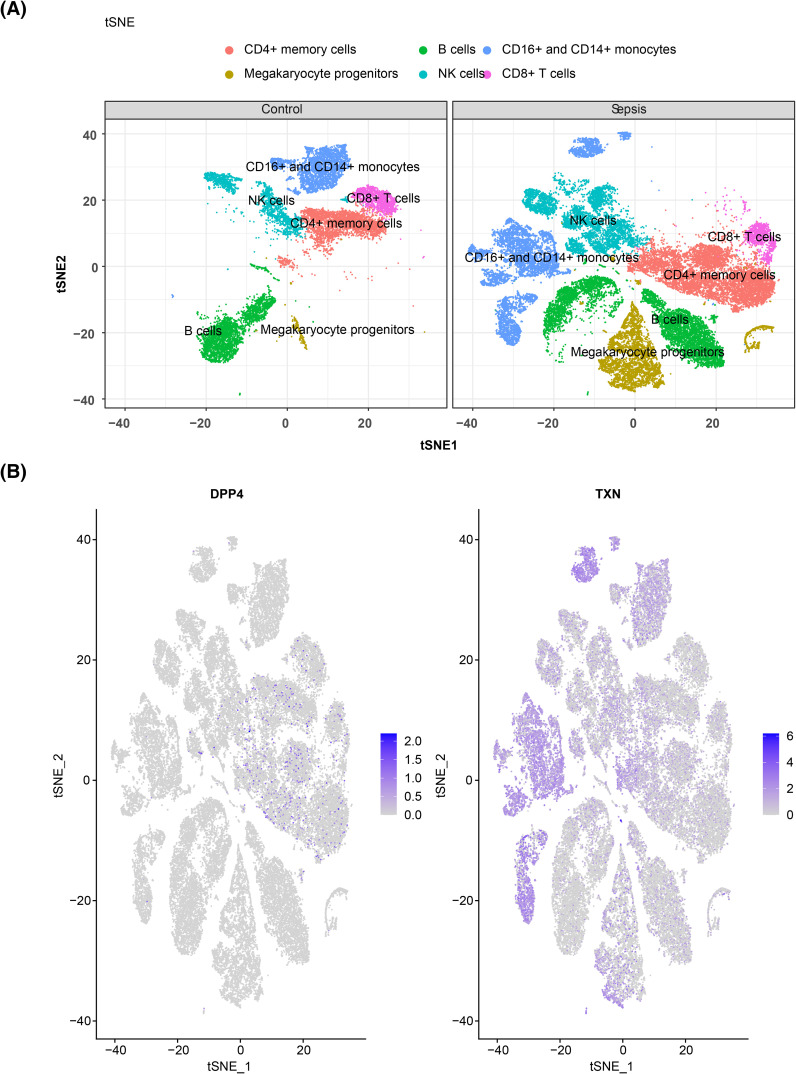
Cell clustering and verification of prognostic gene expression. **(A)** t-SNE clustering diagram of cells in different groups, with the control group of normal cells clustered on the left and the disease group of cells clustered on the right. **(B)** Gene t-SNE plot, with the distribution of the DPP4 gene in immune cells on the left and the distribution of the TXN gene in immune cells on the right. t-SNE, t-distributed stochastic neighbor embedding.

In single-cell analysis, raw data from GSE167363 underwent QC. The pre- and post-QC data are shown in **Supplementary Figures S5A** and **B**, with 45,265 cells and 20,696 genes retained for subsequent analyses. The top 2,000 highly variable genes were identified, including HBB, HBA2, and HBA1 (**Figure 9A**). PCA revealed no obvious outliers (**Figure 9B**), although statistical significance declined after the 30th PC, as reflected in the PC fragmentation curve, leading to the selection of 30 PCs for further analysis (**Figure 9C**, **Supplementary Figure S5C**). The t-SNE clustering grouped the cells into 26 distinct clusters (**Figure 9D**). Using marker gene expression (**Supplementary Figure S5D**), these clusters were annotated into six cell types: B cells, megakaryocyte progenitors, CD4^+^ memory cells, natural killer (NK) cells, CD16^+^ and CD14^+^ monocytes, and CD8^+^ T cells (**Figure 9E**). Notably, megakaryocyte progenitors and CD4^+^ memory cells were predominantly found in the sepsis group (**Figure 10A**). Expression analysis of the biomarkers revealed that DPP4 was primarily expressed in CD4^+^ memory cells, while TXN was predominantly expressed in CD16^+^ and CD14^+^ monocytes, NK cells, CD4^+^ memory cells, and B cells (**Figure 10B**, **Supplementary Figures S5E, F**).

### 3.9 RT-qPCR validation

**Figure 11:**
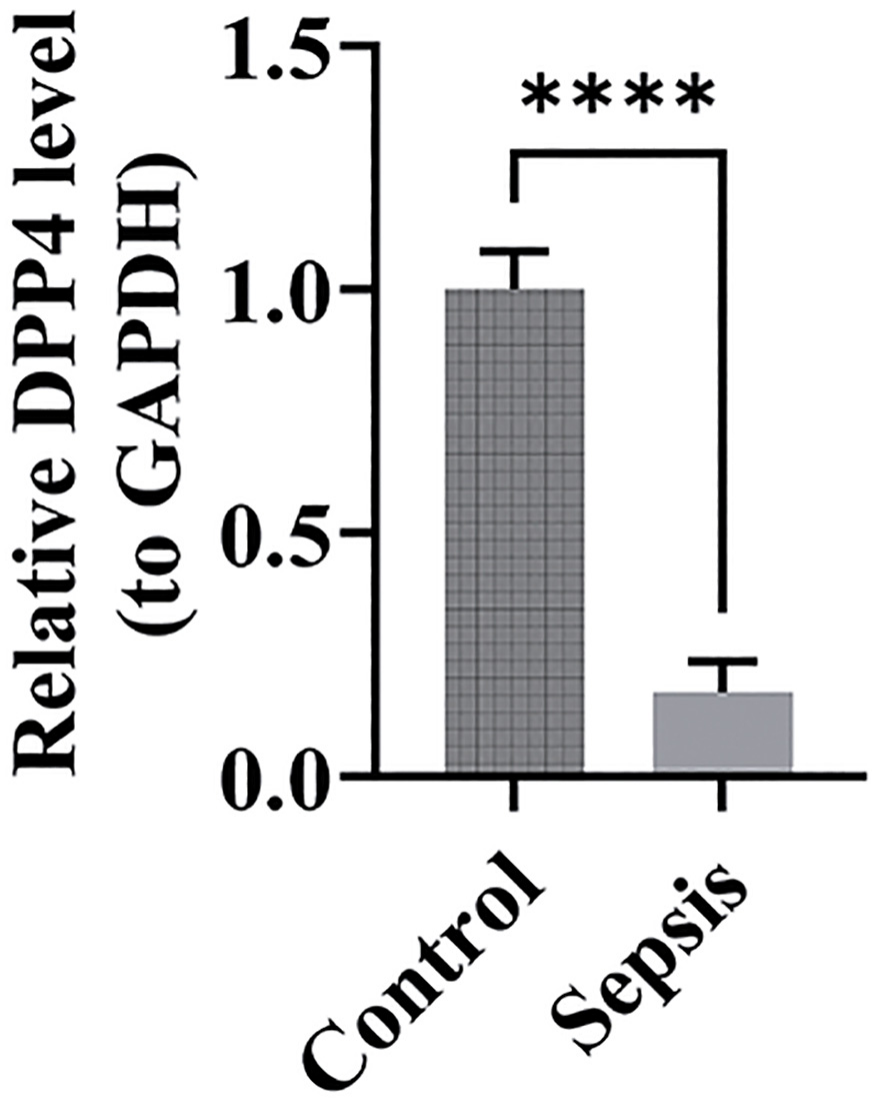
Relative expression bar chart for DPP4. ****, p < 0.0001.

The expression levels of the biomarkers in clinical samples were assessed using RT-qPCR. The results showed significantly higher expression of DPP4 in the control group (**Figure 11**) (p < 0.05), and its expression trend was consistent with the dataset, suggesting that DPP4 has strong diagnostic potential for sepsis and can be effectively validated in clinical settings. However, the expression trend of TXN was opposite to that in the dataset, which may have been due to the small sample size, leading to result bias.

## 4 Discussion

Sepsis is a highly heterogeneous clinical syndrome (1) and remains a leading cause of global morbidity and mortality (40). Despite extensive research efforts over the past decades, the precise pathogenesis of sepsis remains elusive, and both its diagnosis and treatment continue to pose significant challenges. Current therapeutic approaches primarily focus on symptomatic management, including stabilization of hemodynamic parameters, anti-infective treatment, and organ function support. At present, however, there are no specific diagnostic or therapeutic strategies available (41, 42). As sepsis severity escalates, the mortality rate rises accordingly, underscoring the critical importance of timely identification and intervention to improve clinical outcomes. Delayed treatment can markedly impact survival (43). While biomarkers are central to sepsis diagnosis, risk stratification, and prognosis, no definitive marker or therapeutic tool has yet been established. In recent years, the roles of m^6^A methylation and ferroptosis in sepsis have garnered increasing attention.

This study was conducted within this context, aiming to explore the expression profiles and diagnostic potential of FRGs and m^6^A-RGs in sepsis through comprehensive bioinformatics analysis. The goal was to identify potential biomarkers for early diagnosis and therapeutic intervention. Two GEO datasets, GSE65682 (training set) and GSE13904 (validation set), were used to identify 3,755 DEGs, including 1,524 upregulated and 2,231 downregulated genes in sepsis versus control samples. WGCNA was performed on m^6^A-RGs to identify relevant gene modules, which were intersected with FRGs, resulting in 85 candidate genes. Following PPI network analysis, and SVM-RFE and LASSO machine learning, as well as ROC curve validation and expression level verification, two biomarkers—DPP4 and TXN—were identified. These two key biomarkers were then integrated into a nomogram, which illustrated the prediction process and demonstrated predictive accuracy. The nomogram’s performance was further validated using a calibration curve, confirming its robust predictive capability. GSEA revealed that DPP4 and TXN were significantly enriched in pathways such as spliceosome activity and antigen processing and presentation. Additionally, immune infiltration analysis was performed, identifying 24 immune cell types with significantly distinct expression levels between the sepsis and control groups. Correlation analysis between the biomarkers and differentially expressed immune cells was also conducted. To predict potential therapeutic drugs, the Comparative Toxicogenomics Database (CTD) was employed, and network diagrams were constructed to visualize the results. Molecular docking was performed for the top three drugs targeting each biomarker. Finally, the expression profiles of these biomarkers at the single-cell level were investigated, offering valuable insights and novel concepts for the early diagnosis and intervention of sepsis.

A series of bioinformatics analyses identified two biomarkers, DPP4 and TXN. Expression validation revealed that DPP4 was predominantly highly expressed in the control group, while TXN exhibited elevated expression in the sepsis group. DPP4, also known as CD26, functions as a T-cell costimulatory molecule. It is an endogenous type II transmembrane glycoprotein and serine exopeptidase capable of cleaving X-proline dipeptides from the N-terminus of polypeptides. Its diverse substrates are implicated in sepsis (44). DPP4 exists in both membrane-bound and soluble forms (sDPP4), with the latter circulating throughout the body (45). Prior research has shown that sDPP4 can be released from adipose tissue as an inflammatory adipokine, establishing a link between insulin resistance and low-grade inflammation (46). DPP4 plays key roles in glucose and insulin metabolism, as well as immune regulation—processes highly relevant to sepsis. In a nested case–control study by Chia-Jen Shih et al., no significant association was found between DPP4 inhibitor use and sepsis development in hospitalized patients with type 2 diabetes (47). However, other studies suggest that patients with type 2 diabetes starting treatment with SGLT2 inhibitors experience a higher incidence and mortality of sepsis compared to those treated with DPP4 inhibitors (48). Furthermore, some studies have reported a significant reduction in DPP4 expression in patients with sepsis and septic shock (49), while higher DPP4 expression correlates with improved patient survival (50). These findings align with the expression patterns observed in this study, suggesting that DPP4 may serve as a potential prognostic biomarker for sepsis.

TXN is a protein-coding gene involved in various redox reactions, catalyzing disulfide–disulfide bond exchanges through the reversible oxidation of its active site, disulfide, into a disulfide bond (51). The TXN gene regulates B-cell differentiation and function and has been implicated in cardiac damage resulting from severe inflammation (52). Previous research has highlighted TXN’s critical role in inflammation, with ongoing investigations into its therapeutic potential for a variety of diseases (53). Yi Zhou et al. demonstrated that TXN is a unique endoplasmic reticulum-associated gene in sepsis, with significantly upregulated expression in septic rats, positioning it as a potential biomarker for sepsis diagnosis (54). Similarly, TXN was identified as a candidate diagnostic gene for sepsis-induced acute respiratory distress syndrome in a study focused on key iron death genes (55). Additionally, TXN has been recognized as an important differential gene and potential diagnostic marker for early pediatric septic shock compared to healthy children (56). Literature also suggests that inhibiting the pathway mediated by TXN may aid in the treatment of inflammatory diseases (57, 58) and cancer (59–61). In conclusion, TXN has emerged as a central or key gene in sepsis research, consistently showing significant upregulation in sepsis samples, aligning with the findings of this study.

To further investigate the biological roles of the identified biomarkers, correlation analysis and GSEA were performed on the two genes. The correlation analysis revealed a significant negative relationship between TXN and DPP4 (r = −0.43, p < 0.05) (**Figure 5A**). GSEA was then employed to uncover the functional pathways associated with these biomarkers. Both TXN and DPP4 were found to be enriched in several shared pathways, including spliceosome, antigen processing and presentation, primary immunodeficiency, and regulation of autophagy. The spliceosome, a multi-megadalton ribonucleoprotein (RNP) complex composed of five snRNPs and numerous proteins, catalyzes precursor mRNA splicing. During this process, an intricate RNA–RNA and RNP network forms and is reorganized repeatedly to align the pre-mRNA motifs for catalytic processing (62). Previous studies have shown that spliceosome-related pathways are downregulated in the blood of patients with sepsis (63). Immune dysfunction in sepsis often manifests as antigen presentation defects and adaptive immunodeficiencies, which affect T- and B-cell functions (64). Antigen presentation involves the internalization, processing, and peptide binding of antigens to MHC-I molecules, followed by their transport to the cell surface (65). This process is primarily facilitated by monocytes or macrophages, which play key roles in both adaptive immunity and inflammatory modulation in the innate immune response (66). Immunodeficiencies, whether primary or secondary, are a major contributing factor to the progression of sepsis. Prior research has highlighted a link between sepsis and pathways related to primary immunodeficiencies (67). Additionally, abnormal autophagy in macrophages or mitochondria plays a critical role in sepsis pathogenesis (68, 69). Autophagy, which is closely linked to inflammation and immunity, may confer a protective role in sepsis by negatively modulating macrophage activation, altering macrophage polarization, reducing inflammatory vesicle activation and inflammatory factor release, and controlling macrophage apoptosis. However, excessive autophagy may lead to macrophage autophagic death, exacerbating the inflammatory response (70).

We conducted molecular docking for the top 3 drugs with the highest interaction scores from the drug prediction results for two biomarkers. Among the three predicted drugs for DPP4, dutogliptin, an orally effective selective DPP4 inhibitor, failed in docking and may require further experimental validation of its efficacy. Although primarily used for antiviral treatment, valacyclovir exhibited a high binding score (−6.7) with DPP4, suggesting potential cross-interactions. Recent studies have found that valacyclovir can regulate granulocyte-macrophage infiltration by reducing pro-inflammatory cytokines such as TNF-α and IL-6, thereby improving inflammatory responses (71). Alogliptin benzoate, an approved DPP4 inhibitor, had a binding score (−5.3) that supports its interaction with DPP4. Studies have shown that alogliptin reduces leukocyte activation and oxidative stress levels by inhibiting DPP4 activity, significantly improving survival rates in sepsis mouse models (72). In the molecular docking of the three predicted drugs for TXN, PX-12, as a TXN inhibitor, showed a lower Vina score (−3.9), indicating a weaker binding affinity for TXN. Previous studies have shown that PX-12 can act as an antitumor drug by enhancing oxidative stress-induced apoptosis, but its mechanism of action in sepsis requires further exploration (73). Biotinylated gambogic acid and gambogenic acid exhibited higher binding potential (Vina score −6.5 and −6.7), potentially regulating redox balance by targeting TXN. Gambogic acid and its derivatives have been shown to alleviate inflammatory responses in sepsis-induced myocardial injury models by inhibiting the NF-κB pathway (74).

In the development of sepsis, a complex immune response is initiated within the body, which ultimately leads to widespread impairment of cellular function and overall organ dysfunction. Increasing evidence suggests that immune cell infiltration plays a critical role in the pathogenesis of sepsis. In this study, an immune infiltration analysis of 28 immune cell types was performed in both the sepsis and control groups of the training set. Our findings revealed that nine immune cell types were significantly more infiltrated in the sepsis group, while 15 others showed higher infiltration in the control group. Correlation analysis between the two biomarkers (DPP4 and TXN) and the 24 immune cell types that displayed these differential infiltration patterns showed that DPP4 was strongly positively correlated with effector memory CD8 T cells and activated CD8 T cells but negatively correlated with macrophages and activated dendritic cells. DPP4 was also positively correlated with TXN. However, TXN showed a strong negative correlation with both effector memory CD8 T cells and central memory CD4 T cells. In this study, the positive correlation (R > 0.3) between DPP4 and both effector memory CD8 T cells and activated CD8 T cells may reflect the importance of DPP4 in maintaining the phenotypic and functional characteristics of T-cell memory. The long-term survival of effector memory CD8 T cells relies on the homeostasis of metabolic and signaling pathways (41). DPP4 may enhance the survival and function of effector T cells by modulating the activity of relevant chemokines, thereby potentiating the immune response against infections. In sepsis, T-cell exhaustion and immunosuppression are core pathological features (46). High expression of DPP4 may delay immunosuppression by maintaining effector T-cell function, which is consistent with the trend of high DPP4 expression in the control group observed in this study. Macrophages drive the inflammatory response in the early stages of sepsis by releasing pro-inflammatory cytokines such as TNF-α and IL-6. The membrane-bound form of DPP4 can inhibit macrophage activation (50); thus, low DPP4 expression may exacerbate the pro-inflammatory phenotype of macrophages, leading to increased tissue damage. TXN (thioredoxin) positively correlates with macrophages and dendritic cells: TXN is a key regulatory molecule in oxidative stress. High expression of TXN may maintain the survival of macrophages and dendritic cells by scavenging ROS while simultaneously promoting their pro-inflammatory functions (54). In sepsis, this mechanism may contribute to an imbalance of inflammation. TXN influences T-cell metabolism by regulating redox balance. Overactivation of TXN may exacerbate mitochondrial oxidative stress, leading to T-cell apoptosis or functional failure (54). In the late stages of sepsis, T-cell exhaustion serves as a marker of immunosuppression (68). High expression of TXN may accelerate T-cell dysfunction by promoting oxidative damage, which is consistent with the high TXN expression observed in the sepsis group in this study. The ability to develop and maintain memory CD8 T cells following infection or immunization is a hallmark of the adaptive immune response and forms the basis for effective vaccination against infectious diseases (75). However, prior studies have shown that sepsis significantly reduces the number of lymphocytes, including memory CD8 T cells, through apoptosis, resulting in immune paralysis during the early stages of sepsis (76). Additionally, our study identified that the pathways co-enriched with both DPP4 and TXN in GSEA included macrophage autophagy-related pathways. These pathways are upregulated during sepsis, potentially reducing macrophage apoptosis and influencing immune responses. Dendritic cells (DCs) play a central role in the innate immune system, regulating both innate and adaptive immunity (77). DCs are essential for recognizing harmful pathogens, presenting antigens, activating adaptive immunity, and promoting autoimmune immune tolerance, while also having a pro-inflammatory function in the context of sepsis (78).

Subsequent single-cell analysis revealed that DPP4 is predominantly expressed in CD4^+^ memory cells, while TXN is primarily expressed in CD16^+^ and CD14^+^ monocytes. This finding suggests that both biomarkers may contribute to the progression of sepsis through these cell types. CD4^+^ T cells, particularly memory cells, undergo significant depletion during the acute phase of sepsis. This depletion leads to a transient decline in the number of pre-existing memory CD4^+^ T cells, along with sustained dysfunction, which increases susceptibility to secondary infections in sepsis survivors (79). The observed depletion of memory CD4^+^ T cells in patients with sepsis is consistent with the higher expression of DPP4 in these cells, supporting its potential role in sepsis progression. Monocytes, a heterogeneous cell population, are classified into three subpopulations based on the differential expression of CD14 [lipopolysaccharide (LPS) receptor] and CD16 (FcγIII receptor): classical CD14^++^CD16^−^, intermediate CD14^++^CD16^+^, and non-classical CD14^+^CD16^+^ (80). CD14 is a 55-kDa glycosylphosphatidylinositol-anchored receptor that is widely expressed in cells, existing in either cytosolic or secreted protein forms (81). CD14 expression is induced during infectious and inflammatory conditions (82). Recent studies have shown that soluble CD14 isoforms (presepsin) have diagnostic and prognostic values in sepsis (83). The CD16^+^ monocyte subpopulations, characterized by higher pro-inflammatory cytokine production and enhanced antigen presentation ability, are thought to play a key role in sepsis (84). In a study by Guanguan Qiu and colleagues, CD14^++^CD16^+^ (CD16^+^) monocytes were positively correlated with severe sepsis and early disease severity scores in infectious shock (85).

The low expression of DPP4 in sepsis observed in this study aligns with the findings of Vliegen et al. (44), who reported a significant reduction in DPP4 activity in the plasma of patients with septic shock. Additionally, the research by Lambeir et al. (45) supports the crucial role of DPP4 in immune regulation, which concurs with our single-cell analysis showing that DPP4 is primarily expressed in CD4^+^ memory T cells. Regarding TXN, our findings are consistent with those of Zhou et al. (54), who identified a significant upregulation of TXN in a sepsis rat model, suggesting its potential as a diagnostic biomarker. Furthermore, the study by Li et al. (67) also confirms that TXN is a key ferroptosis-related gene in sepsis-induced acute respiratory distress syndrome. This study is the first to reveal the expression patterns of DPP4 and TXN in specific immune cell subsets at the single-cell level. We found that DPP4 is predominantly expressed in CD4^+^ memory T cells, while TXN is highly expressed in CD16^+^ and CD14^+^ monocytes. This discovery provides a new perspective on understanding immune cell dysfunction in sepsis. Notably, CD16^+^ monocytes have been shown by Qiu et al. (85) to positively correlate with the severity of sepsis, which corroborates our findings. This study is the first to report a significant negative correlation between DPP4 and TXN in sepsis (r = −0.43, p < 0.05). This finding offers new insights into the interplay between oxidative stress and immune regulation in sepsis. Although there are currently no direct studies exploring the interaction between DPP4 and TXN in sepsis, the research by Xu et al. (58) indicates that TXN plays a pivotal role in oxidative stress-related diseases, providing indirect support for our observations.

RT-qPCR results in this study demonstrated that DPP4 expression was significantly higher in the control group (p < 0.05), aligning with findings from the database. These results support the hypothesis that DPP4 has strong diagnostic potential for sepsis. Moreover, they validate the true expression of DPP4 in clinical samples, underscoring the reliability of our diagnostic model. Despite the significant upregulation trend of TXN observed in database analysis, its expression trend was inconsistent with the database results in our RT-qPCR validation. This may be the result of the combined effects of multiple factors. First, the sample size used in experimental validation was relatively small, which may not comprehensively and accurately reflect the true expression profile of TXN, thus leading to discrepancies with the database results. In addition, factors such as sample heterogeneity, the timing of sample collection, and the severity of sepsis may also have an impact on the outcomes. Sample heterogeneity: Differences in ethnicity, geography, age, and other aspects may exist between the samples in the database and our clinical samples, potentially leading to variations in gene expression. The training set patients in our study were all European Caucasian adults with an average age of approximately 59 years, the validation set patients were all American Caucasian children with an average age of approximately 2 years, and the patients we validated using RT-qPCR were East Asian adults with an average age of approximately 42 years. The differences in race and age among these three groups are notable. Additionally, studies have indicated that there may be significant differences in immune responses and gene expression regulation among different ethnic groups, which may in turn affect the expression levels of TXN. Timepoints of sample collection: The expression of TXN may vary at different disease stages (e.g., early, middle, and late stages). The samples in the database did not clearly indicate the specific sampling time and the severity of the patients’ conditions, potentially originating from patients at different disease stages, whereas our clinical samples were collected within 4 hours after sepsis diagnosis, which may contribute to the differences in expression levels. Severity of sepsis: The expression of TXN may be influenced by the severity of sepsis. The samples in the database may have included patients with varying degrees of severity, whereas our clinical samples focused on the early stage of sepsis, which could also lead to differences in expression levels. Since the severity of sepsis was not clearly indicated in the training and validation datasets, we were unable to compare it with the severity of the patients we validated. In the future, we will further validate the expression of TXN by expanding the sample size to include a more diverse patient population, encompassing patients with varying severity of sepsis, diverse demographic characteristics, and a broader range of underlying diseases, as well as by optimizing experimental conditions. Additionally, we will explore its potential role in sepsis.

In this study, a comprehensive analysis of m^6^A-RGs and FRGs was conducted in sepsis using publicly available data, yielding several valuable insights. Nevertheless, certain limitations should be acknowledged. Although this study has revealed the diagnostic value of DPP4 and TXN in sepsis and their correlation with immune infiltration through bioinformatics methods, the specific molecular mechanisms still require further experimental validation. For instance, the high expression of TXN in CD16^+^ monocytes may affect the progression of sepsis by regulating the release of pro-inflammatory cytokines or the ferroptosis pathway, while the absence of DPP4 expression in memory CD4^+^ T cells may exacerbate immune suppression. Future studies will combine animal models and functional experiments to directly validate the functions of these biomarkers in sepsis and their regulatory pathways. Second, although this study conducted RT-qPCR to validate the expression of biomarkers, there remains a lack of functional experimental validation for DPP4 and TXN. Therefore, we plan to directly verify the roles of DPP4 and TXN in the pathogenesis of sepsis through gene knockout or overexpression experiments in the future and to explore their specific molecular mechanisms. In addition, we intend to combine *in vitro* cell models and animal experiments to further investigate the functions of DPP4 and TXN in pathways such as immune regulation and oxidative stress in order to gain a comprehensive understanding of their roles in sepsis. Simultaneously, we will also confirm the reliability of the transcriptome data through protein-level validation (such as Western blotting), further strengthening the functional and regulatory mechanisms of these biomarkers in sepsis and providing more direct evidence for future targeted therapies. The data utilized in this study were sourced from public databases. While these databases provide abundant transcriptome data, their heterogeneity may impact the research findings. Specifically, there are significant differences in the subject populations between the training and validation sets, with the training dataset primarily derived from adult populations and the validation dataset from pediatric populations. Variations among studies may stem from differences in sample processing, sequencing platforms, and data analysis methods, which can affect the accuracy and reliability of the results. To enhance the reliability of our findings, we plan to more carefully select and integrate higher-quality, multicenter, and multiethnic public datasets in the future. By implementing standardized preprocessing procedures, including uniform data cleaning, format conversion, and other operations, we aim to reduce data heterogeneity arising from technical differences. Randomized splitting will be conducted to minimize the impact of heterogeneity on the results and to validate the generalization ability of the model. Additionally, we will collect more samples encompassing various types, targeting different populations (such as adults/children, different races) and disease stages (such as varying severities of sepsis). Stratified analysis or mixed data resampling techniques (such as cross-validation) will be employed to assess the generalization ability of the model, thereby improving the broad applicability of the research conclusions and enhancing the representativeness and robustness of the study Furthermore, we conducted drug prediction and molecular docking to explore the issue of targeted therapy modified by the biomarkers we screened. However, the aforementioned drugs still need to be validated and their mechanisms of action explored in sepsis *in vitro* models. Various concentrations of these drugs should be tested to assess their inhibitory effects on DPP4/TXN, as well as their impacts on immune cell function, apoptosis, and inflammatory cytokines. Through experiments on cell proliferation, cytokine release, immune cell infiltration, and other aspects, we aim to verify the potential therapeutic effects of these compounds in sepsis models. This investigation will continue to examine the roles of m^6^A methylation and ferroptosis while advancing novel research methods and approaches to provide more precise and actionable insights for sepsis diagnosis and treatment. Additionally, broader participation in this field is encouraged to further deepen and expand sepsis research.

The translation of the findings on DPP4 and TXN in this study into clinical practice can be approached from the following aspects. Potential directions for diagnostic tool development—rapid test kit development: Based on the differential expression of DPP4 and TXN in the blood (with DPP4 highly expressed in the control group and TXN highly expressed in the sepsis group), we plan to collaborate with *in vitro* diagnostic companies to develop rapid test kits using ELISA or microfluidic technology. For instance, by detecting decreased DPP4 concentrations or increased TXN concentrations in patient sera, combined with clinical scores [such as the Sequential Organ Failure Assessment (SOFA) score], the early diagnostic efficiency for sepsis can be enhanced. Similar strategies have been successfully applied in the clinical detection of procalcitonin (PCT) and presepsin (83). Dynamic monitoring and prognostic assessment: The high expression of TXN is associated with oxidative stress and immunosuppression, and its dynamic changes may reflect patients’ responses to treatment. In the future, multi-timepoint sampling can be used to assess the correlation between TXN levels and organ dysfunction (such as acute respiratory distress syndrome) or mortality, providing a basis for individualized treatment (55). Translational potential of therapeutic strategies—preclinical validation of targeted drugs: Molecular docking results indicate that gambogenic acid (targeting TXN) and valacyclovir (targeting DPP4) have high binding potential. We plan to validate the efficacy of these drugs in sepsis mouse models, such as those induced by intraperitoneal injection of LPS or cecal ligation and puncture (CLP), observing their impact on inflammatory cytokines (e.g., TNF-α and IL-6) and survival rates. Studies have shown that the DPP4 inhibitor alogliptin can improve survival rates in sepsis mouse models (72), providing indirect support for the drug predictions in this study. Exploration of immunomodulatory therapy: Single-cell analysis reveals high expression of DPP4 in CD4^+^ memory T cells, which are significantly depleted in sepsis. Future research can investigate the regulatory role of DPP4 on memory T-cell function through *in vitro* experiments (e.g., T-cell coculture), exploring its potential as an immune checkpoint molecule.

## 5 Conclusions

Two biomarkers, DPP4 and TXN, were identified and validated in the context of sepsis. Immune infiltration and therapeutic potential were also assessed at the single-cell level, offering new perspectives for sepsis treatment. Based on the expression characteristics and molecular mechanisms of DPP4 and TXN, future research will focus on 1) developing rapid detection kits, 2) validating the efficacy of targeted drugs, and 3) exploring immunomodulatory strategies. Additionally, multicenter cohort studies and functional experiments will lay the foundation for further translation.

## Data Availability

Publicly available datasets were analyzed in this study. This data can be found here: GSE65682 (https://www.ncbi.nlm.nih.gov/geo/query/acc.cgi?acc=GSE65682), GSE13904 (https://www.ncbi.nlm.nih.gov/geo/query/acc.cgi?acc=GSE13904), GSE167363 (https://www.ncbi.nlm.nih.gov/geo/query/acc.cgi?acc=GSE167363).

## Supplementary material

Supplementary Figure 1 WGCNA sample clustering and module correlation analysis results. **(A)** Sample clustering plot, where each branch represents a sample and the ordinate represents the height of the hierarchical clustering. The red horizontal line represents the cutoff height for removing outlier samples. **(B)** Heatmap illustrating the correlation between module eigengenes and m6A-RGs scores.

Supplementary Figure 2 Graph of the GO enrichment analysis. The height of the bar graph in the inner circle represents the significance of the term, and the color represents the z-score, with darker colors indicating higher scores. The outer circle shows a scatter plot of the expression level of each gene in each term. Red represents up-regulation and blue represents down-regulation.

Supplementary Figure 3 The changing characteristics of each variable coefficient in Lasso regression.

Supplementary Figure 4 Immune infiltration levels of 28 immune cells in the training set.

Supplementary Figure 5 The results of single-cell dataset-related analysis. **(A)** Violin plots of nFeature_RNA, nCount_RNA and percent_mt before single-cell data quality control. **(B)** Violin plots of nFeature_RNA, nCount_RNA and percent_mt after single-cell data quality control. **(C)** Principal component inflection point diagram. **(D)** Cell marker dot plot. **(E)** Gene violin plot, with the distribution of the DPP4 gene in immune cells. **(F)** Gene violin plot, with the distribution of the TXN gene in immune cell.

Supplementary Table 1 Ferroptosis-related genes (FRGs).

Supplementary Table 2 m6A-related genes (m6A-RGs).

Supplementary Table 3 GO enrichment analysis of the candidate genes.

Supplementary Table 4 22 KEGG pathways.

Supplementary Table 5 Four candidate biomarkers.

Supplementary Table 6 GSEA identified the enriched pathway of TXN and DPP4.

Supplementary Table 7 Primer sequences.

## Glossary

m^6^A: *N* ^6^-methyladenosine
m^6^A-RGs: m^6^A-related genes
FRGs: ferroptosis-related genes
DEGs: differentially expressed genes
WGCNA: weighted gene co-expression network analysis
ROC: receiver operating characteristic
RT-qPCR: reverse transcription–quantitative PCR
WHO: World Health Organization
ROS: reactive oxygen species
scRNA-seq: single-cell RNA sequencing
GEO: Gene Expression Omnibus
PBMCs: peripheral blood mononuclear cells
ssGSEA: single-sample gene set enrichment analysis
ME: module eigengene
GO: Gene Ontology
KEGG: Kyoto Encyclopedia of Genes and Genomes
BP: biological process
MF: molecular function
CC: cellular component
STRING: Search Tool for the Retrieval of Interaction Gene/Proteins
LASSO: least absolute shrinkage and selection operator
SVM-RFE: support vector machine recursive feature elimination
AUC: area under curve
HL: Hosmer–Lemeshow
GSEA: Gene Set Enrichment Analysis
NES: normalized enrichment score
FDR: false discovery rate
MSigDB: Molecular Signatures Database
DGIdb: Drug–Gene Interaction Database
PDB: Protein Data Bank
PCA: principal component analysis
PCs: principal components
t-SNE: t-distributed stochastic neighbor embedding
cDNA: complementary DNA
DCs: dendritic cells
