# Supplementary figures and images for: Identification and validation of m6A RNA methylation and ferroptosis-related biomarkers in sepsis: transcriptome combined with single-cell RNA sequencing
Source: Front Immunol. 2025 Mar 7;16:1543517. doi: 10.3389/fimmu.2025.1543517 (PMC11925765; doi:10.3389/fimmu.2025.1543517)

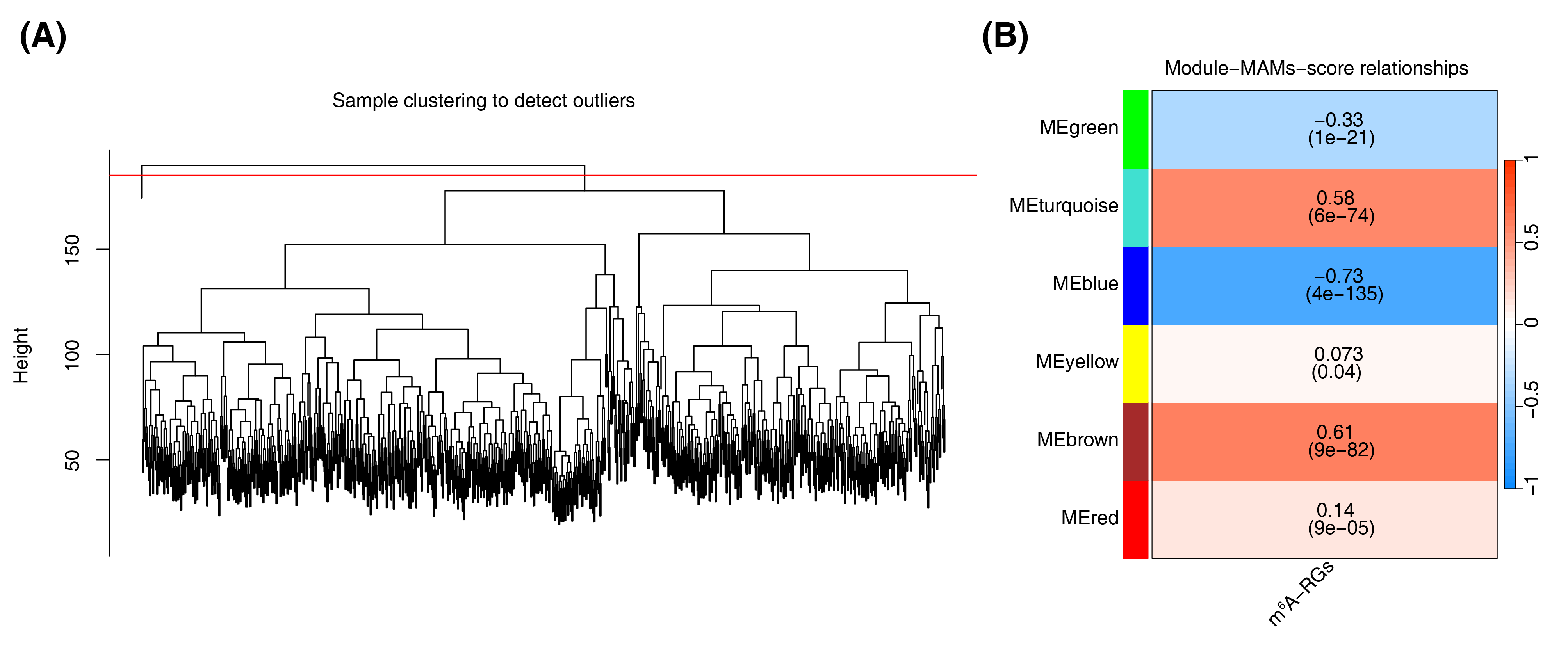

Supplement: Supplementary Figure 1 — WGCNA sample clustering and module correlation analysis results. (A) Sample clustering plot, where each branch represents a sample and the ordinate represents the height of the hierarchical clustering. The red horizontal line represents the cutoff height for removing outlier samples. (B) Heatmap illustrating the correlation between module eigengenes and m6A-RGs scores. [file Image1.tif]

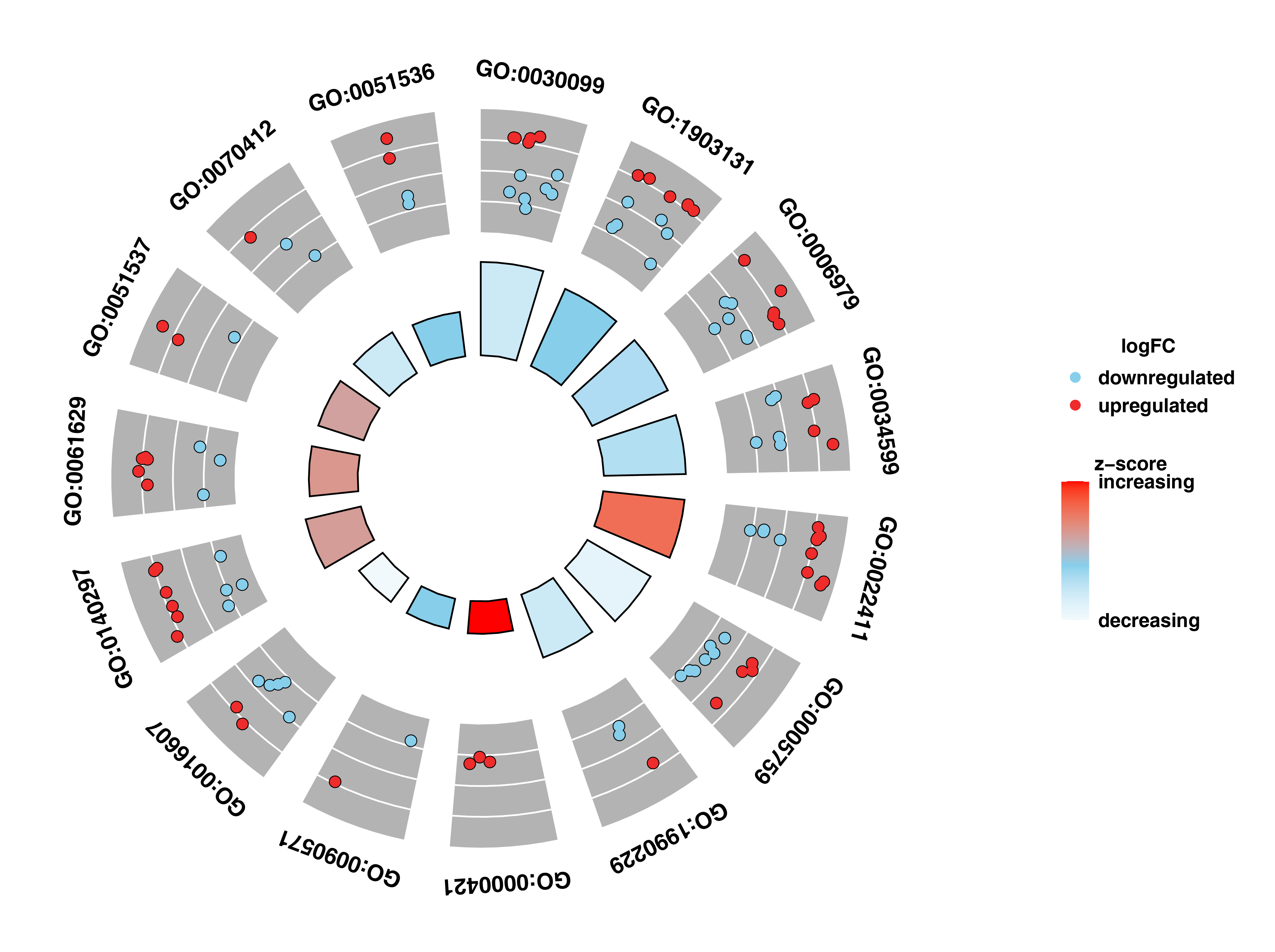

Supplement: Supplementary Figure 2 — Graph of the GO enrichment analysis. The height of the bar graph in the inner circle represents the significance of the term, and the color represents the z-score, with darker colors indicating higher scores. The outer circle shows a scatter plot of the expression level of each gene in each term. Red represents up-regulation and blue represents down-regulation. [file Image2.tif]

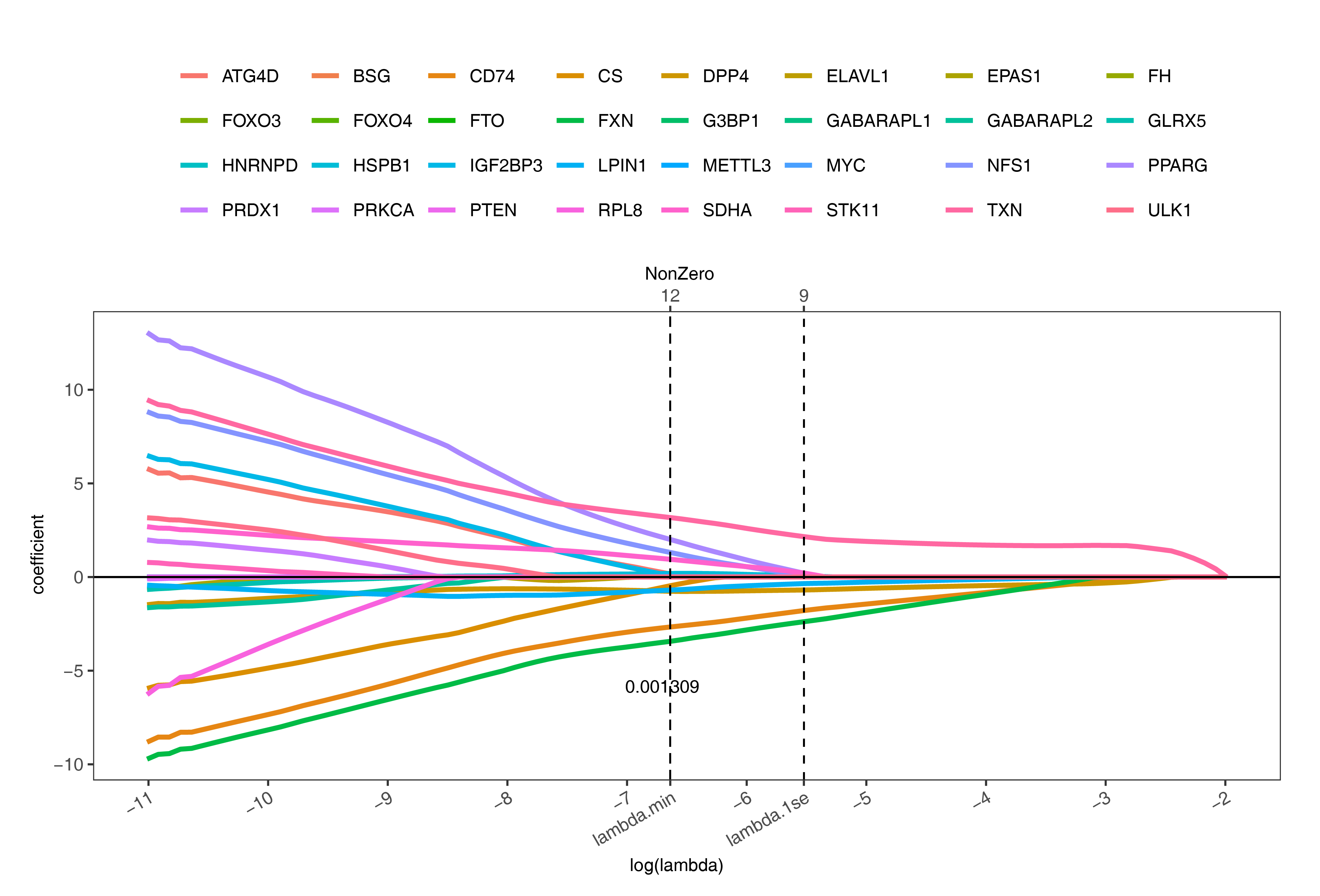

Supplement: Supplementary Figure 3 — The changing characteristics of each variable coefficient in Lasso regression. [file Image3.tif]

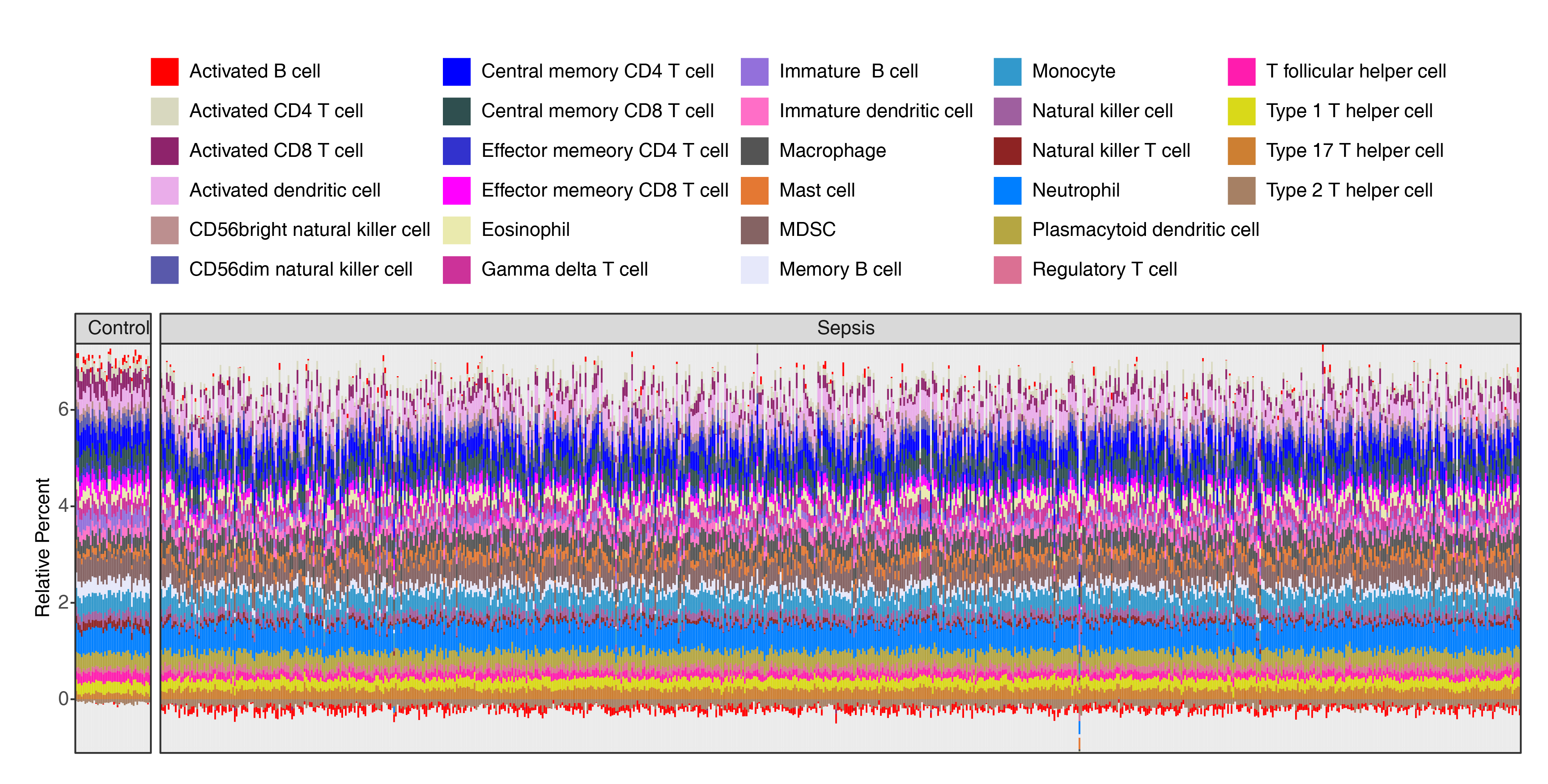

Supplement: Supplementary Figure 4 — Immune infiltration levels of 28 immune cells in the training set. [file Image4.tif]

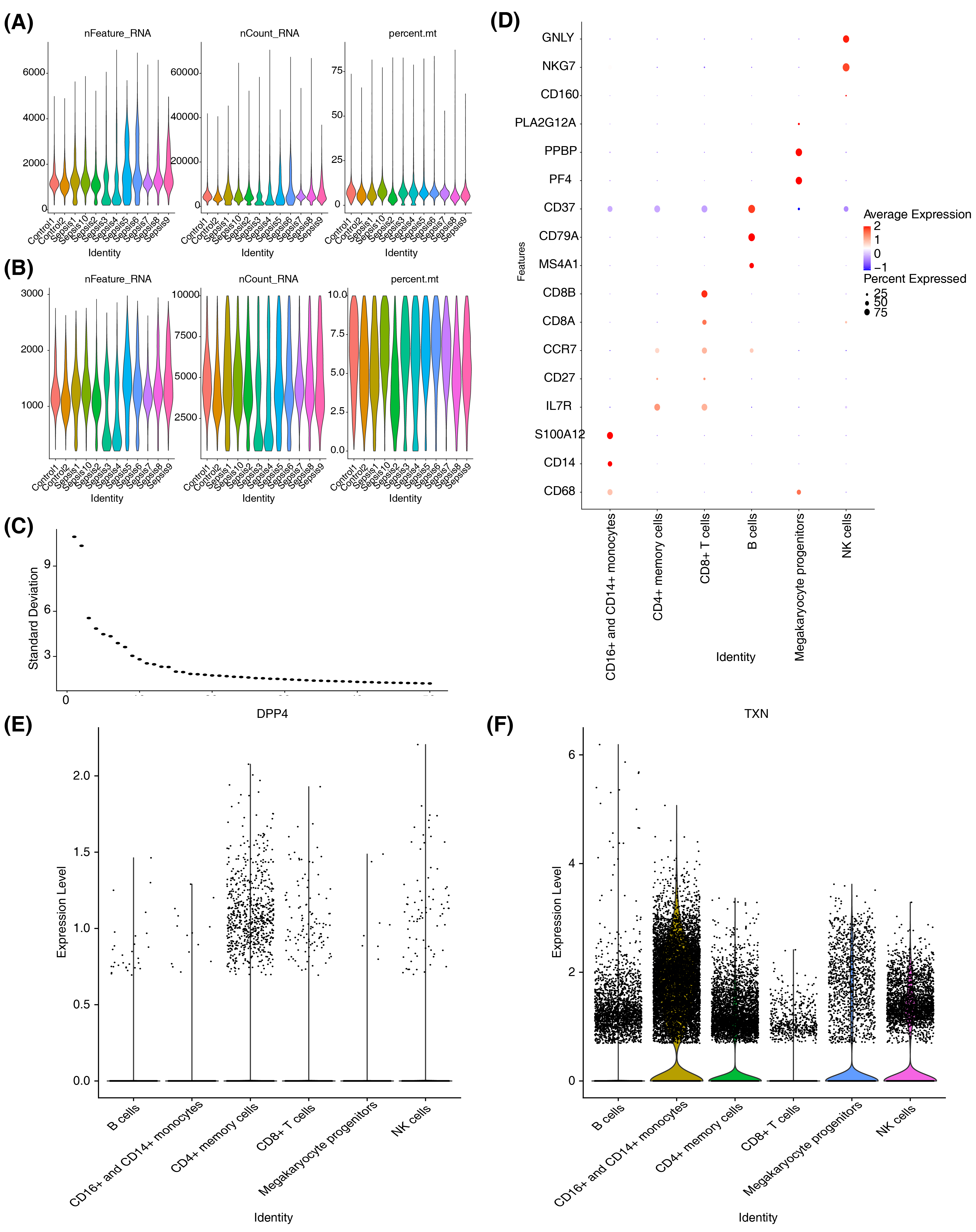

Supplement: Supplementary Figure 5 — The results of single-cell dataset-related analysis. (A) Violin plots of nFeature_RNA, nCount_RNA and percent_mt before single-cell data quality control. (B) Violin plots of nFeature_RNA, nCount_RNA and percent_mt after single-cell data quality control. (C) Principal component inflection point diagram. (D) Cell marker dot plot. (E) Gene violin plot, with the distribution of the DPP4 gene in immune cells. (F) Gene violin plot, with the distribution of the TXN gene in immune cell. [file Image5.tif]
