# Supplementary material for: Identification and validation of m6A RNA methylation and ferroptosis-related biomarkers in sepsis: transcriptome combined with single-cell RNA sequencing
Source: Front Immunol. 2025 Mar 7;16:1543517. doi: 10.3389/fimmu.2025.1543517 (PMC11925765; doi:10.3389/fimmu.2025.1543517)
Supplement: Supplementary Table 7 — Primer sequences. [file Table7.docx]

| **Primer** | **Sequences** | |
| --- | --- | --- |
| TXN F | CTTGGACGCTGCAGGTGATA | |
| TXN R | TCCTGACAGTCATCCACATCT |  |
| DPP4 F | ATGCCAGGAGGAAGGAATCT |  |
| DPP4 R | TCCAGGACTCTCAGCCCTTT |  |
| Internal reference-GAPDH F | CGAAGGTGGAGTCAACGGATTT |  |
| Internal reference-GAPDH R | ATGGGTGGAATCATATTGGAAC |  |

Table S7 Primer sequences
